# The non-market value of reclaiming natural landscape and biodiversity: a Dutch case study

**DOI:** 10.1007/s10113-025-02418-5

**Published:** 2025-06-09

**Authors:** Peter John Robinson, Marjolijn van Schendel, Jeroen C. J. H. Aerts, Wouter Botzen, Pieter van Beukering

**Affiliations:** Department of Environmental Economics, Institute for Environmental Studies, VU University Amsterdam, De Boelelaan 1087, 1081 HV Amsterdam, The Netherlands

**Keywords:** Biodiversity, Choice experiment, Non-market value, Landscape, Nature-inclusive farming

## Abstract

Biodiversity and natural landscapes have been lost over time due to global agricultural expansion and urbanization. Our study assesses the non-market economic value of reclaiming natural landscapes in the Zuid-Limburg region of the Netherlands, a country in which large-scale intensive agriculture dominates rural landscapes. Through a discrete choice experiment conducted among non-residents of Zuid-Limburg, we find that individuals planning to visit the area are willing to contribute a similar (insignificantly different) monetary amount toward conservation efforts in the area as those who do not plan to visit. This implies that the area is largely valued for its indirect use values and non-use values rather than its option values. We further find that societal concerns explain some preferences in the choice experiment. In particular, increased levels of concern about the Dutch nitrogen crisis relate to a higher preference for agricultural businesses to be contributing to nature-inclusive farming. More concern about built-up area leads to a higher preference for natural landscapes. Furthermore, individuals with more concern about extinction risk prefer fewer animal species in Zuid-Limburg with threatened status. Moreover, our study reveals that individuals with greater patience and higher household incomes are more willing to invest in conservation efforts.

**Supplementary Information:**

The online version contains supplementary material available at 10.1007/s10113-025-02418-5.

## Introduction

Natural land-cover changes, i.e. changes to the extent of natural landscapes, are a driver of biodiversity decline. Agricultural expansion is an especially important cause of this decline (Davison et al. 2021; IPBES 2018). Alongside rapid urbanization in the past three decades, agricultural expansion has come at the expense of forest, natural grassland^1^ and wetland areas (IPBES 2019). It is also projected that demand for agricultural goods will rise far into the future, which means that agricultural expansion is likely to remain a global threat to natural landscapes well into the future (Kastner et al. 2012; Tilman et al. 2011; Zabel et al. 2019). Here and throughout this paper, we refer to natural landscapes as areas with a focus on conserving or restoring natural ecosystems, as opposed to agricultural or urban areas.

Economic valuation studies, that aim to derive the benefits or costs of changes to natural landscapes in monetary units, can serve a number of purposes for environmental conservation. For instance, these studies may be used to raise awareness about how biodiversity and natural landscapes contribute to human welfare, as well as assist in the prioritization of conservation actions (Costanza et al. 1997). They can also inform policymaking decisions about interventions that result in land-cover change, such as changing urban or agricultural zones into natural landscapes, via societal cost–benefit analyses (Gorissen et al. 2020; Nunes and van den Bergh 2001; Ring et al. 2010; van Wensem 2013; Wainaina et al. 2020; Atkinson and Mourato 2008).

A literature review was conducted of peer-reviewed DCE economic valuation studies that examine preferences for changes to the extent or existing composition of natural landscapes (Table A1, Online Resource 1). The review was conducted to identify the knowledge gap related to our research interest of examining residents’ preferences for land-cover changes toward natural landscapes. The reviewed studies were subcategorized by country of investigation, landscape types and sample. The majority of these studies examine one specific landscape type among general populations of residents. Most are also conducted within Europe; however, only a few have been implemented in the Netherlands. One such study by Koetse et al. (2017) did not attempt to value reclaiming natural landscape in an existing region. Instead, they use a generic experiment design in which respondents faced choice alternatives that do not refer to a specific study site. They aimed to provide value estimates for natural landscapes with different characteristics that can be transferred to alternative study areas in the Netherlands. Another study by van Zanten et al. (2016) examines the aesthetics of different types of agricultural landscapes in the Netherlands. They investigate whether individuals prefer to see small forest patches in these landscapes. However, we do not know whether their respondents would have preferred another land-cover type instead. They also did not elicit the monetary welfare effects of natural landscape changes. To the best of our knowledge, no study has yet examined the non-market value of transforming an existing region of the Netherlands with non-natural landscapes into various types of natural landscapes. Non-market values are values contributing to human well-being that are not traded on markets, and therefore do not have a price.

This paper assesses the non-market economic value Dutch society derive from the conversion of agricultural and urbanized areas into additional forest, natural grassland and wetland areas, as well as higher levels of fauna biodiversity, via a discrete choice experiment (DCE) conducted among Dutch residents. In our study, we examine preferences for biodiversity based on the number of animal species assigned threatened status, which specifically focuses on the abundance of animals within particular groups of species.

Our study contributes to the existing (non-DCE) landscape valuation literature in the Netherlands, which has tended to examine use values in isolation, i.e. benefits extracted from human interactions with natural landscapes, such as tourism and the amenity value of living close to natural environments, e.g. for recreational and aesthetic benefits, among other indirect uses (Remme et al. 2014; 2015; Horlings et al. 2020; Hein 2011). Indirect use values are specific types of non-market use values provided by environmental resources to individuals without them directly interacting with or consuming these resources, e.g. carbon sequestration.

Non-use values attached to the existence of biodiversity in the Netherlands, and its preservation for future generations, have largely been neglected to date. Non-use values are non-market values derived from either the existence or the potential future use of environmental resources, even if they are not currently being used. The adoption of Natural Capital Accounting (NCA) consistent approaches for eliciting economic values in previous Dutch studies has restricted the elicitation of non-use values. NCA methods utilize exchange values, whereas non-use values must be derived using welfare-based stated preference approaches like DCEs. The need to estimate both exchange values and welfare benefits has been highlighted by the United Nations (2017) and Turner et al. (2019) to support specific policy and decision-making contexts.

We separate values for those who reside outside our study area, and in the future plan to visit the area, from those who do not plan to visit the area. By doing so, we differentiate between those who derive less tangible landscape conservation benefits, i.e. indirect use and non-use (e.g. existence and altruistic) values, and individuals who derive, in addition, more tangible benefits via option (direct use) values (e.g. through recreation) (Birol and Cox 2007; Do and Bennett 2009; Hanley et al. 1998; Mallawaarachchi et al. 2001). Option values refer to non-use values of preserving environmental resources for the possibility of future use, e.g. when residents visit an area. In addition to the landscape focus of our study, we showcase public support for the role of agriculture in maintaining and enhancing biodiversity via nature-inclusive farming methods.

The remainder of this paper is structured as follows: the “Case study: Zuid-Limburg” section describes the case study area. The “Methods” section outlines the methods applied, including the way in which the DCE was implemented and the econometric specification adopted for statistical modelling as well as our hypotheses. The “Results” section elaborates on the main results and the “Discussion” section discusses these findings and highlights some key policy implications. The “Conclusion” section concludes the paper.

## Case study: Zuid-Limburg

The area focus of our study is Zuid-Limburg, a region of the Limburg province located in the south of the Netherlands. Zuid-Limburg is a distinct geographical area with rolling hills and valleys shaped by the River Meuse and its tributaries Geul and Geleenbeek. The region has more than 600,000 inhabitants and is surrounded by the so-called Euregio Maas-Rijn, which includes the large urban areas of Liege (Belgium) and Ruhrgebiet (Germany).

Most of the area in Zuid-Limburg is currently covered by some form of agriculture that is dominated by mixed dairy and crop farming as well as fruit trees (34,796 hectares, 52.7%) (Fig. A1, Online Resource 2). Urban area covers 20,408 hectares (30.9%). Furthermore, 5063 hectares (7.7%) can be classified as forest or natural grassland and 4818 hectares (7.3%) as pastures. Water (922 hectares, 1.4%) and wetland area (57 hectares, 0.1%) are also present.

After World War II, to raise food production and to adapt farm fields to mechanized agriculture, a number of land consolidation projects were implemented in Zuid-Limburg at the expense of landscape features like natural terraces and woodland (Spaan et al. 2010). In addition, due to rising population numbers and the mining industry, at the beginning of the twentieth century, there was a considerable expansion of urban area (Duijsings and Napier 1994). However, following the establishment of the Nature Network Netherlands (NNN) in 2013, whose overarching objective is to prevent further natural landscape and biodiversity decline, the Province of Limburg plans to convert 2600 hectares of non-natural landscapes back to natural landscapes by 2027 (Provincie Limburg 2022). Given the high current policy relevance of natural landscape and biodiversity changes, it is relevant to study societal preferences for these changes.

Furthermore, following a ruling of the European Court in 2018, the State Council of the Netherlands called for a drastic reduction in nitrogen emissions from intensive livestock farming (that is often located close to protected areas) (Stokstad 2019; Erisman 2021a; 2021b). Since the ruling in 2018, there has been large-scale civil unrest and polarization of opinions with respect to the nitrogen crisis and the actions proposed by the government to reduce emissions, especially in the agriculture sector (Otjes and de Jonge 2023). Targets set by the Dutch government mean that in Limburg as a whole nitrogen emission must be more than halved, while in Natura 2000 protected natural landscapes emissions must be reduced by at least 95%, which calls for more sustainable farming solutions among other measures.

## Methods

### Implementation of the discrete choice experiment and survey

A web-based survey embedded a DCE on preferences of the general public in the Netherlands for natural landscape conservation in Zuid-Limburg.^2^ Six attributes were included in the DCE with the purpose of generating realistic scenarios of the current situation and the effect of conservation efforts, on the basis of which respondents could choose whether to contribute money toward conservation measures. The first DCE attribute, the number of threatened species, refers to the number of animal species characteristic of Zuid-Limburg that is in some form threatened with extinction. The species that were included in the DCE were selected according to the classification of National Landscape Zuid-Limburg that indicated these species are characteristic of the area. The species were grouped based on their current status of conservation within the Dutch target species list. In sum, 17 species were classified based on the International Union for Conservation of Nature (IUCN) criteria as being critically endangered, endangered or vulnerable (Online Resource 3). To define the attribute, an upper bound (best-case scenario) was considered where 15 species would be taken off the threatened list with additional conservation, followed by 10 species, 5 species and no change to the number of threatened species (status quo scenario).

To examine respondents’ preferences for land-cover changes, three different land-cover attributes were included in the DCE, i.e. an increase or no change to forest coverage, natural grassland coverage, and wetland coverage, because of the contribution potential to natural landscape values of these land-cover classes. For all three land-cover classes, the same levels were chosen, i.e. + 500 hectares, + 200 hectares, + 100 hectares and no change, as the increases correspond to realistic transformations for Zuid-Limburg according to the planned change of 2600 hectares for the province as a whole. We communicated these hectare changes to the respondents in terms of soccer pitch equivalents in order to provide reference values that are easier to grasp cognitively. In the instructions prior to the DCE, the respondents were informed that to make room for new natural landscape area the same area would be removed from pastures, urban area and arable area in equal proportions. We kept the land-cover changes comparable for the different attributes because we were interested in which changes (to extent) individuals may value the most. Furthermore, in our opinion specifying different land-cover changes would have substantially increased the cognitive load on the respondents, who already had to process the different hectare changes in football field equivalents.

The fifth attribute indicates the proportion of farming businesses that incorporate some form of nature-inclusive farming. Nature-inclusive farming integrates management of natural resources by farmers and ensures ecological functions and biodiversity on and around farms. It was communicated to the respondents that farmers can contribute to nature-inclusive farming in a number of ways, including adopting grass variety, reducing pesticide and fertilizer use, restoring natural landscape elements around farms, and reducing noise while mowing. According to Nature-inclusive Agriculture Limburg, in 2022, 50% of the farmers in Limburg were active in nature-inclusive farming, which suggests they were adopting at least some nature-inclusive farming practices. They aim for this percentage to grow to 80% (+ 30 percentage points) by 2025 and to 100% (+ 50 percentage points) by 2030. These future scenarios were included as levels in the DCE to test individuals’ preferences toward nature-inclusive farming in Zuid-Limburg by assuming similar changes would occur there as for the province as a whole, where the 2022 situation is included as the status quo scenario.

The final attribute is a yearly contribution to conservation that the respondents would pay in terms of a municipal tax increase per household. Municipal taxes are levied separately from national income taxes and are very familiar for Dutch residents. Five levels of tax increase are included. A summary of attributes and their corresponding levels can be found in Table 1.

**Table 1:**
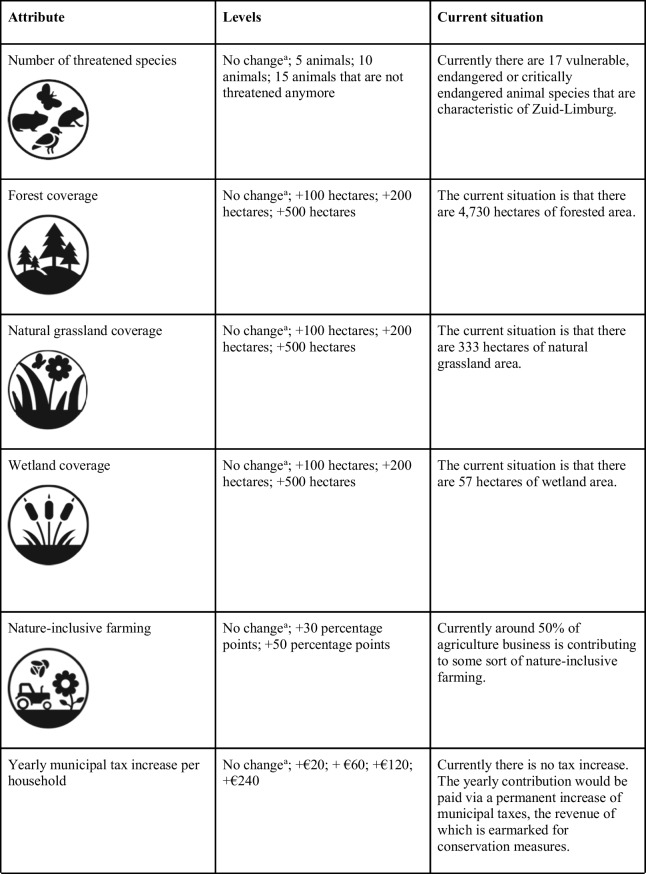
List of attributes and their corresponding levels within the discrete choice experiment

Note: ^a^Attribute levels assigned to the business-as-usual (status quo) scenario

One problem that may arise in DCE studies is that of perceived correlations among attributes that may lead the respondents to think there are unacceptable combinations of attributes within the experiment design (Hensher et al. 2005). We do not think this is a problem in our case given the way we communicated the attribute changes. More specifically, it was explained to the respondents that measures can be taken to increase the size of natural landscapes, biodiversity and the number of farms applying nature-inclusive farming methods, e.g. through protection and restoration activities, transformation of existing land into natural landscapes and direct funding for farmers to implement nature-inclusive farming practices. It was further communicated to the respondents that the changes can be supported by the higher municipal taxes, the revenues of which are used to pay for the abovementioned measures. We did not communicate to the respondents that more or less money (or effort) would go to one particular measure. Therefore, we believe that the way in which the (causes of the) attribute changes were described to the respondents suggests that all the choice experiment scenarios are reasonable, including all scenarios of land-cover and biodiversity change. Furthermore, the pre-test showed that the respondents had no problems with finding the experiment unrealistic.

The DCE was programmed in Sawtooth Software Lighthouse Studio version 9.4, and distributed in October and November 2022 among a random sample of 1503 Dutch residents via the online survey panel of Panel Inzicht (https://panelinzicht.nl/). Note that the residents of Zuid-Limburg that are included in the sample are not included in the analysis of this paper. The respondents were required to be aged 18 years or over to participate. Prior to the DCE, the respondents received information about current land-cover in Zuid-Limburg,^3^ the current state of animal species characteristic for the area that are either vulnerable, endangered or critically endangered, information about farmers incorporating nature-inclusive farming methods currently, and instructional text that explained the various choices that the respondents would face in the DCE (see Online Resource 2 for an overview of the DCE instructions).

The respondents were asked to choose between three options that were defined by the number of threatened species, forest coverage, natural grassland coverage, wetland coverage, nature-inclusive farming and the yearly municipal tax increase per household. Only one of each of the attribute levels was presented per option. Two of the options (A and B) represent scenarios based on additional conservation measures funded by the revenue earned by raising municipal taxes. The third option (C) reflects a “business-as-usual” scenario in which there is no additional conservation effort. Each respondent faced six unique choice sets that were drawn at random from ten different versions of the DCE that were used in a blocked design.

A D-efficient statistical design generated by Ngene software was applied for the versions of the DCE with priors based on expectations of the signs of coefficients on each attribute. We favor this design to an orthogonal design in order to omit dominated alternatives and to achieve a high level of statistical precision with respect to estimated choice model coefficient estimates (Rose et al. 2008). An example choice set can be found in Online Resource 4.

Prior to the DCE, we included a “cheap talk script” by asking the respondents to “consider carefully how much extra money you can afford to spend each year to contribute to the conservation of Zuid-Limburg’s nature”. Moreover, we omit 44 individuals from our sample who specifically indicated that they made random choices in the choice experiment tasks based on the follow-up question: “How did you make your choices?”.

Using survey questions, we elicited respondents’ age in years, gender, education level, household income, levels of concern about a range of societal issues and time preferences, as well as whether they have visited Zuid-Limburg in the past 10 years or plan to visit there in the future (see Online Resource 5) for the specific questions that were used to elicit these variables and how they were coded for the analysis. Note here that time preferences refer to the willingness of individuals to delay gratification for a potentially higher future reward. Individuals with higher discount rates tend to be less willing to wait for future rewards, and individuals with lower discount rates are more willing to delay gratification, i.e. they are more patient. Time preferences were elicited with a widely adopted question (Falk et al. 2018) modified to incorporate a sliding scale. We incorporated the sliding scale to increase the level of granularity regarding the data for this variable.

### Econometric specification

To account for potential heterogeneity in DCE choice behaviour across individuals, we estimate a Panel Mixed Logit (ML) model,^4^ where coefficients of interest are allowed to vary over respondents. The coefficient vector ${\gamma }_{i}$ is the sum of the population mean variable weights, $b$, and an individual deviation from the mean, ${\eta }_{i}$. This deviation can take any distributional assumption, and is measured by its standard deviation. According to random utility maximization theory (McFadden 1974), the utility of a choice alternative $j$ for individual $i$ can be expressed as:

1 $${V}_{ij}=b{Z}_{ij}+{{\eta }_{i}Z}_{ij}+{\varepsilon }_{ij}$$

where ${Z}_{ij}$ is a vector of observed variables, including the choice attributes, and ${\varepsilon }_{ij}$ is the random error component of utility that is independent and identically distributed with a type-1 extreme value distribution. For the ML models we apply, that include the choice attributes only, the following utility function is specified:

2 $$\begin{array}{l}{V}_{ij}=ASC+{\beta }_{1}{Number \;species \;off\; threatened status}_{ij}+{\beta }_{2}{Forest \;coverage}_{ij}+\\ {\beta }_{3}{Wetland \;coverage}_{ij}+{\beta }_{4}{Nature-inclusive\; farming}_{ij}+\\ {\beta }_{5}{Yearly \;municipal \;tax \;increase \;per \;household}_{ij}+{\varepsilon }_{ij}\end{array}$$

ASC is the alternative-specific constant, where the no additional conservation effort (business-as-usual) alternative is dummy coded as one. The land-cover attributes are coded as hectare changes multiplied by one hundred,^5^ and the nature-inclusive farming attribute represents percentage point changes, relative to the business-as-usual scenario. A normal distribution for the random parameters is used for these attributes and the ASC. Furthermore, the municipal tax increase attribute, which is coded in Euros per year, is assumed to have a linear fixed effect on utility to permit the calculation of welfare estimates, as is common practice in choice modelling analysis (Hensher et al. 2001). Marginal effects are calculated for changes to the attributes and can be defined as the impact of a one-unit increase in an attribute on the probability of choosing to pay for conservation. The ML models are estimated using 1000 Halton draws.

Mean WTP (Euros) per year is calculated for changes in the attribute levels, i.e. for every species removed from threatened status, one hundred hectare changes for the land-cover attributes and percentage point changes to the proportion of agriculture business contributing to nature-inclusive farming. WTP is derived by taking the negative ratio of the utility function attribute parameters to the cost (tax increase) parameter, which is a widely used practice for DCE choice models (Hensher et al. 2005). Statistical significance is derived using the Krinsky and Robb (1986) bootstrapping method with 1000 random draws.

### Hypotheses

Individuals who reside outside of a particular region, and have no intention of visiting that region in the future, may still value the implementation of conservation measures in the area for altruistic reasons or because they generally value the continued existence of ecosystems in good condition (Johansson-Stenman 1998). These individuals may also derive indirect use values in the form of carbon sequestration benefits (Remme et al. 2015). Furthermore, individuals who do plan to visit the region may additionally extract option values from conservation measures that, for instance, preserve the option of experiencing the area’s natural beauty at a later date (Ghosh and Mondal 2013). We hypothesize that individuals who plan to visit Zuid-Limburg are willing to pay more for conservation measures there, because they enjoy a wider diversity of values, compared to individuals who do not plan to visit Zuid-Limburg.

- **H1**: Individuals who plan to visit Zuid-Limburg in the future are willing to pay more for conservation measures there, compared to individuals who do not plan to visit Zuid-Limburg.

We also expect time preferences to be influential in our setting, since conservation decisions generally amount to a choice of whether to incur certain costs now in return for (uncertain) future benefits (Bernedo and Ferraro 2017). That is, individuals who heavily discount the future are likely to be less willing to pay for conservation efforts resulting in long-term ecological improvements, compared to individuals with lower discount rates (Fehr and Leibbrandt 2011).

- **H2**: Individuals who have high discount rates are willing to pay less for conservation measures, compared to individuals who have lower discount rates.

We expect household income to influence the amount individuals are willing to pay for conservation measures as well, since this affects their ability to pay for such measures. Stated preference environmental valuation studies typically find that willingness-to-pay (WTP) is strongly related to either individual or household income levels (Robinson et al. 2022; Baskaran et al. 2009; Huang et al. 2018).

- **H3**: Individuals who have high levels of household income are willing to pay more for conservation measures, compared to individuals who have lower household income levels.

Moreover, we expect concerns about various threats to the environmental attributes to explain preferences toward these attributes, consistent with theories of threat perception and attitude change (Loewenstein et al. 2001). In the context of our DCE, concerns about the Dutch nitrogen crisis could explain why individuals prefer more agricultural businesses adopting nature-inclusive farming methods, which can tackle nitrogen levels (Vermunt et al. 2022). Furthermore, concerns about built-up area and extinction risk may influence preferences for the levels of natural landscape and the number of threatened animal species, respectively, as these issues of concern can directly lead to changes to these levels.

- **H4**: Individuals who have high levels of concern about the nitrogen crisis prefer more agricultural businesses to be contributing to nature-inclusive farming, compared to individuals who have low levels of concern about this issue.
- **H5**: Individuals who have high levels of concern about an increase to levels of built-up area prefer more natural landscape, compared to individuals who have low levels of concern about this issue.
- **H6**: Individuals who have high levels of concern about extinction risk prefer fewer animal species with threatened status, compared to individuals who have low levels of concern about this issue.

For **H1**, we conduct Poe et al. (1997; 2005) independent samples combinatorial tests to investigate whether there are significant differences in WTP between individuals residing outside Zuid-Limburg who plan to go there in the future and those who do not plan to go there. To test **H2** and **H3**, we examine the signs and significance levels of coefficient estimates on interaction terms between the tax increase attribute and time preferences and income, respectively. We test **H4**, **H5** and **H6** by assessing the signs and significance levels of coefficient estimates on interaction terms between the nature-inclusive farming attribute and concern about the nitrogen crisis, the land-cover attributes and concern about built-up area, and the number of threatened species attribute and concern about extinction risk.^6^

## Results

### Descriptive statistics

Descriptive statistics for socio-demographic variables in our analyzed dataset as well as whether the respondents have visited Zuid-Limburg in the past 10 years or plan to visit there in the future are presented in Table A3 (Online Resource 5).^7^ The descriptive statistics refer to individual-specific variables elicited among our survey respondents, whereas the attributes refer to variables generated by our experimental design that are therefore presented separately in Table 1.

In the sample, the average age among the respondents is 48.3 years, which is higher than the Dutch average of 42.4 years as reported by the Central Bureau of Statistics (https://www.cbs.nl/), but may be explained by the required minimum age of 18 years to participate in the study. Our data, which consists of 47% male respondents, is close to representative of the gender balance in the Netherlands, where men account for 49.7% of the total population.^8^ The subgroup of individuals in the sample who have completed higher education (university/college graduates or higher) is 31%, which is a little lower than the Dutch average of 35.5%. The average income before tax among households in the sample is between €2000 and €3999 per month, which is also lower than the estimated €6250 per household per month as estimated by the Central Bureau of Statistics for 2022.

In addition to the general socio-demographic variables, our survey derived a number of other factors that may explain choice behaviour in the DCE, such as whether individuals have visited Zuid-Limburg, concerns about several societal issues and short- vs. long-term time preferences. Table A3 shows that 91% of the respondents have visited Zuid-Limburg, and 65% of the sample is planning to visit there in the future. Moreover, 22%, 25% and 32% of the respondents are not concerned or do not think about the extinction of animal and/or plant species, the increase in built-up area and the nitrogen crisis, respectively. Fifty-eight percent, 58% and 53% of the respondents are a little bit or moderately concerned and 20%, 17% and 14% are seriously concerned about these societal issues, respectively. Regarding time preferences (patience), on average the respondents tend to be willing to give up something that is beneficial today in order to benefit in the future.

### Regression results

Table 2 displays the choice modelling results of the DCE data based on ML specifications. Model 1 is a pooled model that includes data from all non-residents of Zuid-Limburg. Model 2 adds a series of interaction terms between (1) the number threatened species attribute and concern about extinction; (2) the land-cover attributes and concern about an increase to built-up area; (3) the nature-inclusive farming attribute and concern about the nitrogen crisis; and (4) the yearly municipal tax increase attribute and income as well as time preferences (patience).

**Table 2:** Panel Mixed Logit models

	**Model 1**	**Model 2**	**Model 3**	**Model 4**
Alternative-specific constant (ASC)	− 2.419*** (0.198)	− 2.322*** (0.177)	− 2.428*** (0.225)	− 2.109*** (0.357)
Number species off threatened status	0.063*** (0.005) *0.017*	− 0.038** (0.015)	0.063*** (0.006) *0.018*	0.064*** (0.008) *0.017*
Forest coverage (one hundred hectares)	0.037*** (0.013) *0.003*	− 0.072* (0.041)	0.033** (0.015) *0.003*	0.048** (0.024) *0.004*
Natural grassland coverage (one hundred hectares)	0.026** (0.013) *0.002*	− 0.013 (0.043)	0.016 (0.016)	0.056** (0.024) *0.004*
Wetland coverage (one hundred hectares)	0.044*** (0.013) *0.004*	0.039 (0.043)	0.039** (0.016) *0.003*	0.048* (0.025) *0.004*
Nature-inclusive farming (percentage points)	0.002 (0.002)	− 0.031*** (0.006)	0.003 (0.003)	0.001 (0.004)
Yearly municipal tax increase per household (Euros)	− 0.011*** (0.000) − *0.026*	− 0.020*** (0.001)	− 0.010*** (0.001) − *0.025*	− 0.014*** (0.001) − *0.029*
***Interaction effects***				
Number species off threatened status × concern extinction		0.029*** (0.004)		
Forest coverage (one hundred hectares) × concern built-up area		< 0.001*** (0.000)		
Natural grassland coverage (one hundred hectares) × concern built-up area		< 0.001 (0.000)		
Wetland coverage (one hundred hectares) × concern built-up area		< 0.001 (0.000)		
Nature-inclusive farming (percentage points) × concern nitrogen crisis		0.010*** (0.002)		
Yearly municipal tax increase per household (Euros) × Income		< 0.001*** (0.000)		
Yearly municipal tax increase per household (Euros) × Patience		< 0.001*** (0.000)		
***Standard deviations***				
Alternative-specific constant (ASC)	4.626*** (0.227)	4.032*** (0.203)	4.213*** (0.263)	5.418*** (0.443)
Number species off threatened status	0.075*** (0.008)	0.076*** (0.008)	0.077*** (0.009)	0.071*** (0.014)
Forest coverage (one hundred hectares)	0.162*** (0.027)	0.174*** (0.026)	0.147*** (0.034)	0.173*** (0.049)
Natural grassland coverage (one hundred hectares)	0.180*** (0.026)	0.201*** (0.026)	0.178*** (0.032)	0.178*** (0.047)
Wetland coverage (one hundred hectares)	0.167*** (0.027)	0.183*** (0.027)	0.167*** (0.032)	0.193*** (0.053)
Nature-inclusive farming (percentage points)	0.042*** (0.003)	0.040*** (0.003)	0.043*** (0.003)	0.037*** (0.005)
**Pseudo R-squared**	0.322	0.331	0.297	0.378
**Number respondents**	1297	1297	843	454

Notes: ***, ** and * significance at 1%, 5% and 10% level, respectively. Coefficient estimates are provided with standard errors in parentheses. Marginal effects are provided for significant attributes of attributes only models in italicized text

Models 3 and 4 conduct the same regression analysis as Model 1, separately for individuals who reside outside Zuid-Limburg and plan to go there in the future and for those who do not plan to go there in the future, respectively. It is our assumption that the choices of the latter subgroup are indicative of indirect use and non-use (e.g. existence and altruistic) values. The reason is that the benefits they derive do not depend on any current or future planned direct use.

The results suggest that higher numbers of animal species removed from threatened status induce positive changes to utility. This effect is fairly robust regardless of planned visitation to Zuid-Limburg. Moreover, individuals who are more concerned about extinction prefer more animal species removed from threatened status according to the significant interaction effect between this concern and the attribute.

There is some heterogeneity regarding the impact of the land-cover attributes based on planned visitation. Namely, overall, and for the subgroup of respondents who plan to visit Zuid-Limburg, hectare increases to wetland and forest coverage appear to be valued more than higher levels of natural grassland coverage, whereas among those who do not plan to visit Zuid-Limburg, improvements to natural grassland result in greater utility changes, compared to improved forest and wetland coverage. The following section will explicitly test whether the difference in landscape preferences based on planned visitation can be considered statistically significant. Individuals who are concerned about increased levels of built-up area prefer more forest coverage based on the interaction analysis.

Furthermore, the main attribute effect of the proportion of agriculture business contributing to nature-inclusive farming on choice behaviour is overall not significant. The standard deviation estimate for the nature-inclusive farming attribute reveals that, of all the attributes, preference heterogeneity for this attribute is the most significant. Concern about the nitrogen crisis is also positively correlated with a preference for more agriculture business contributing to nature-inclusive farming based on the interaction analysis.

The coefficient estimate on the yearly municipal tax increase attribute shows that respondents dislike high tax levels to pay for conservation. The interaction analysis implies that individuals with higher household incomes and patience are more willing to support conservation through higher taxes.^9^

Concerning the negative ASC coefficient estimates, overall respondents chose options A and B, representing scenarios of additional conservation, over the third option (C), that reflects the status quo (business-as-usual) scenario in which no extra money is contributed to conservation, more often.^10^ There is also a high level of preference heterogeneity in our sample as displayed with the ASC standard deviation estimate. Significant preference heterogeneity is apparent for the land-cover and animal species attributes in all choice models as well.

### Welfare estimates

**Fig. 1:**
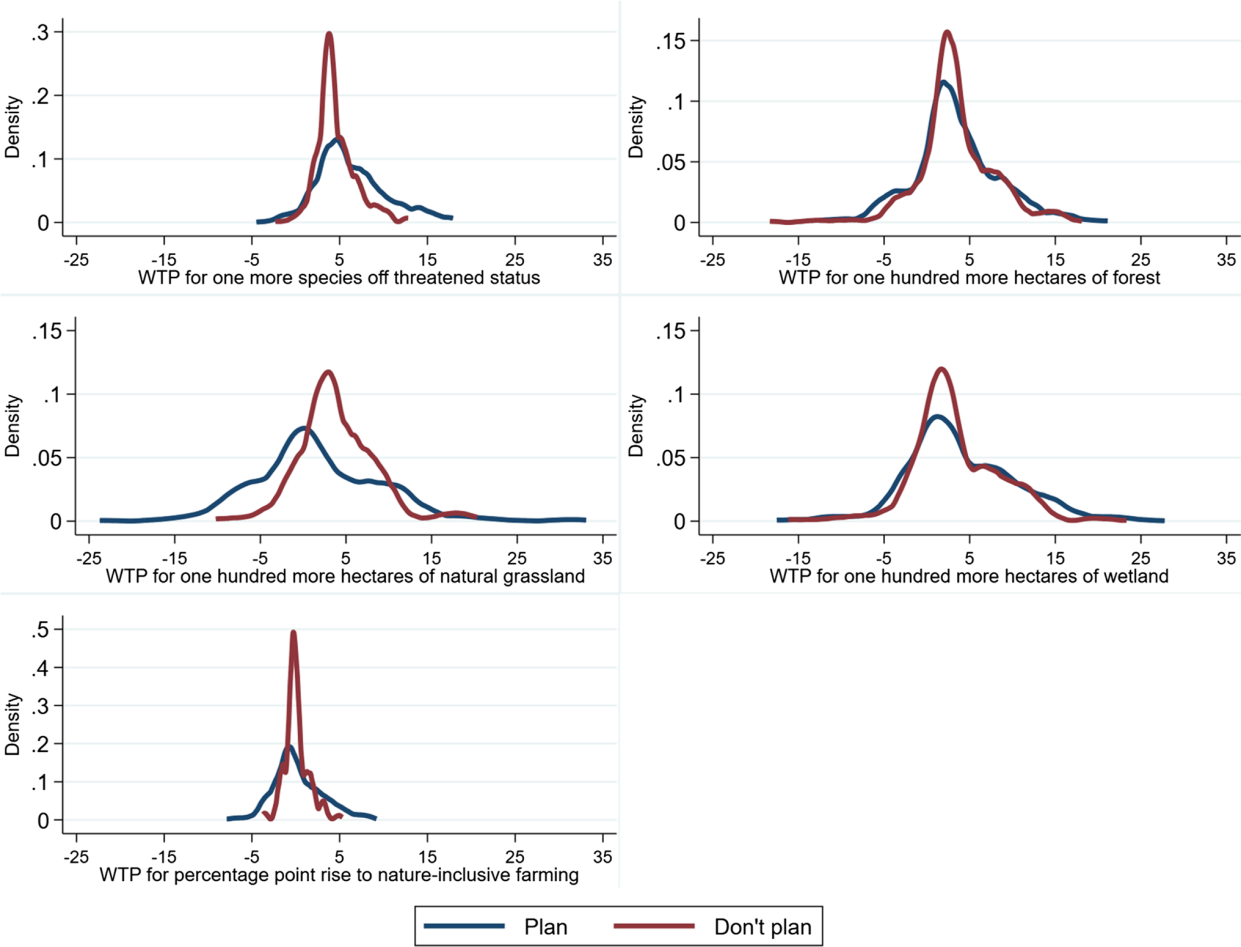
Distribution of willingness-to-pay (WTP) (Euros) for changes to attributes. Notes: *p*-values are obtained from two-sided Poe et al. (1997; 2005) independent samples combinatorial tests of differences between distributions of WTP of individuals who plan and do not plan to visit Zuid-Limburg: number species off threatened status (0.566), forest coverage (0.995), natural grassland coverage (0.630), wetland coverage (0.917) and nature-inclusive farming (0.977)

Figure 1 displays Kernel density estimations of predicted individual WTP values for individuals who plan to go to Zuid-Limburg in the future and for those who do not plan to go there based on the choice modelling results. In Table A4 (Online Resource 6), we measure welfare based on Models 1, 3 and 4 of the previous section.

There are no statistically significant differences in WTP for any of the attributes depending on planned to visitation to Zuid-Limburg (*p*-values > 0.1). The pooled findings show that overall respondents are willing to pay €5.65 per year for the removal of a single animal species from threatened status. Regarding the land-cover attributes, overall respondents are willing to pay a little more per year for one hundred hectare increases to forest coverage (€3.34) and wetland coverage (€3.93), than a one hundred hectare increase to natural grassland coverage (€2.33).

In an additional analysis, we restricted our sample and estimated WTP for respondents who visited Zuid-Limburg in the past for reasons of “outdoor adventure”, “walking” or “cycling”. These reasons may be indicative of the types of activities linked to natural landscapes they may carry out in the future there. The results of this additional analysis can be summarized as follows: Individuals who plan to visit Zuid-Limburg and partook in activities linked to natural landscapes there in the past are not willing to pay more or less toward conservation efforts in the area, compared to those who do not plan to visit Zuid-Limburg (*p*-values = 0.389 (the number of species off threatened status), 0.997 (forest coverage), 0.786 (natural grassland coverage), 0.890 (wetland coverage) and 0.972 (nature-inclusive farming)). The same conclusion holds if we restrict the subgroup of individuals who do not plan to visit Zuid-Limburg to those who never partook in the activities linked to natural landscapes there in the past (*p*-values = 0.393 (the number of species off threatened status), 0.887 (forest coverage), 0.902 (natural grassland coverage), 0.785 (wetland coverage) and 0.943 (nature-inclusive farming)).

In another analysis, we examined whether there is a difference in WTP between respondents who plan to visit Zuid-Limburg compared to those who do not, depending on income. Among individuals with household monthly income levels above the sample median (€2000 to €3999), those who plan to visit Zuid-Limburg are not willing to pay more or less toward conservation efforts in the area, compared to those who do not plan to visit (*p*-values = 0.647 (the number of species off threatened status), 0.886 (forest coverage), 0.822 (natural grassland coverage), 0.904 (wetland coverage) and 0.943 (nature-inclusive farming)). The same conclusion holds if we restrict our sample to individuals with household monthly income levels at or below the sample median (*p*-values = 0.604 (the number of species off threatened status), 0.921 (forest coverage), 0.511 (natural grassland coverage), 0.850 (wetland coverage) and 0.968 (nature-inclusive farming)).

## Discussion

Agricultural expansion and urbanization in recent decades have led to the global loss of biodiversity and natural landscapes (Dudley and Alexander 2017; IPBES 2019). This study assesses the WTP of Dutch residents for reclaiming natural landscapes in Zuid-Limburg, the southernmost region of the Netherlands. Our study is of current policy relevance given that the Province of Limburg plans to convert non-natural landscape areas to natural landscape areas by 2027 (Provincie Limburg 2022). That is, preferences for reclaiming natural landscapes elicited in our DCE can inform Dutch policy makers on the types of landscape restoration that individuals prefer the most. Our choice modelling findings show that individuals residing outside Zuid-Limburg prefer to increase the extent of natural landscapes in the region. Overall, there is a stronger preference for expanding forest and wetland coverage than for expanding natural grassland coverage, which can inform policymakers on the types of natural landscapes citizens prefer most.

Since WTP for changes to natural landscape cover and the removal of animal species from threatened status does not vary significantly based on planned visitation to the area (in contrast to **H1**), it appears that a large share of the value extracted may be non-use (e.g. existence and altruistic), or indirect use (e.g. in the case of forests and wetlands providing carbon sequestration benefits). These values could feature in evaluations of land coverage policymaking decisions for the area, in addition to other use benefits elicited for the Netherlands in previous studies (Remme et al. 2014; 2015; Horlings et al. 2020). However, note that given the low income of our sample compared to the Dutch population, the WTP estimates must be treated with caution and as conservative estimates.

Our finding that planned visitors are unwilling to pay a significant premium for landscape conservation, over those who do not plan to visit Zuid-Limburg, is in contrast to some studies conducted in other countries, where (option) direct uses were identified as a driver of choice behaviour (Birol and Cox 2007; Do and Bennett 2009). Nevertheless, non-use contributions to value can be substantial, as evidenced by previous studies showing that individuals who are not familiar with an area are still willing to pay for environmental improvements there (Hanley et al. 1998), as well as studies finding that non-use values represent the largest share of WTP for conservation of nature (Kontogianni et al. 2012).

Aside from the insignificant difference in WTP based on planned visitation to Zuid-Limburg, our respondents have a clear preference for the creation of new natural landscape and more biodiversity overall. However, we also find that there are heterogeneous preferences between respondents for changes to the individual choice attributes. Our interaction analysis shows that some of this heterogeneity can be explained by concern about increased levels of built-up area, for the forest coverage attribute (supporting **H5**), and concern about species extinction, for the number of threatened species attribute (supporting **H6**).

Apart from preferences for natural landscapes and biodiversity, assessing the current preferences of citizens for nature-inclusive farming can inform policy decisions on whether action is needed. More effective governance to ensure nature-inclusive farming is implemented more broadly may require a change of citizens’ preferences (Runhaar 2017). Our pooled model results show that there is a subgroup of our respondents who prefer more nature-inclusive farming in Zuid-Limburg, and a subgroup who prefer less, given the highly significant standard deviation estimate for this attribute. Our model that includes interaction terms between the choice attributes and individual characteristics shows that concern about the nitrogen crisis explains some of the heterogeneity regarding preferences for nature-inclusive farming (supporting **H4**). These results are aligned with the observed varying opinions concerning topics like the nitrogen crisis and measures farmers can take to tackle nitrogen levels in the Netherlands (Otjes and de Jonge 2023). Education among the general public on nitrogen pollution may be needed to shift public concerns about the nitrogen crisis and to enhance support for nature-inclusive farming. Note that for science education to be effective, it has been suggested that communication may be channeled through group settings among individuals with diverse characteristics and storytelling (Toomey 2023).

With respect to individual-specific factors that may drive WTP, we show that respondents with higher incomes and patience are willing to pay higher tax levels to fund conservation as expected (supporting **H2** and **H3**). The latter result is in line with other studies on sustainable investment decisions, e.g. the planting of mangroves (Boonmanunt et al. 2020), conservation of coral reefs (Robinson et al. 2023) and the protection of common-pool resources (Fehr and Leibbrandt 2011). The relationship between individuals’ time preferences and environmental conservation preferences can guide policymaking. Framing conservation efforts in terms of shorter term benefits, like job opportunities, and improved air and water quality (compared to, e.g., carbon sequestration), could make conservation initiatives more attractive (Hardisty and Weber 2009).

## Conclusion

This paper has examined the stated preferences of Dutch residents for more natural landscapes, animal biodiversity and agriculture business adopting nature-inclusive farming methods in the area of Zuid-Limburg. Our findings are based on a DCE conducted among individuals residing outside the region, which is informative for the elicitation of indirect use and non-use values. In sum, we find that planned visitors to Zuid-Limburg do not differ significantly in their landscape conservation preferences, compared to those who do not plan to visit the area. A higher level of concern about built-up area is associated with a greater preference for natural landscapes. Furthermore, more concern about species extinction is related to a greater preference for animal biodiversity. An increased level of concern about the nitrogen crisis in the Netherlands is associated with a greater preference for the proportion of agricultural businesses contributing to nature-inclusive farming. Moreover, higher household income and patience levels are associated with a greater WTP for conservation. On the basis of these findings, we suggest several possible implications for policymaking, i.e. education and environmental problem description framing interventions. The effectiveness of these potential policy interventions could be examined in future research.

## Supplementary Information

Below is the link to the electronic supplementary material.

Supplementary file1 (PDF 1991 KB)

## Data Availability

The survey and discrete choice experiment datasets analyzed during the current study are available from https://github.com/prn690/zlimburg.

## Footnotes

1 Natural grasslands are under no or very little human influence as per the Corine Land Cover database classification.

2 A pre-test was conducted initially to refine the different elements of the survey text and DCE. The survey follows guidelines of the research ethics committee in the faculty of the university the research was implemented.

3 The geographic areas of forests, natural grasslands, wetlands, arable land, pastures and urban areas were calculated using Geographic Information Systems (GIS) data from the Corine Land Cover database of the Netherlands. The sizes in hectares were communicated to the respondents and the proportions of each land-cover category were shown in a map.

4 The time subscript is suppressed here.

5 The hectare changes are multiplied by one hundred to obtain coefficient estimates that can be presented at a reasonable number of decimal places.

6 In further analyses, we included other variables in interaction analyses based on our interests and prior expectations of possible predictors of individuals’ preferences for levels of the different attributes. We were specifically interested in whether political ideology may affect preferences for agricultural businesses to adopt nature-inclusive farming methods. We included interaction terms between the nature-inclusive farming attribute and political ideology, as measured by conservative versus liberal leaning, as well as whether individuals vote for the Farmer–Citizen Movement political party. The coefficient estimates attached to these other interaction terms were found to be insignificant (*p*-values > 0.1). We also included interaction terms between the attributes and other issues of concern not included in our hypotheses. For instance, we conducted interaction analyses to investigate whether preferences for the land-cover attributes may be explained by concern about urbanization as well as concern about the intensification of agriculture. We further examined whether preferences for nature-inclusive farming could be explained by concern about pollution, concern about climate change and concern about the intensification of agriculture. The concern variables we anticipated might explain preferences for each of the choice attributes are highly correlated with the concern variables already included in our core hypotheses of these preferences. Adding these concern variables to our analyses led to unstable coefficient estimates and inflated standard errors. We do not report details of the further analyses in the “Regression results” section which had a high risk of multicollinearity and model complexity.

7 We can only infer option values depending on whether individuals plan to visit the area.

8 We applied quotas for age and gender. We used age categories (18 to 34 years, 35 to 49 years, 50 to 64 years and 65 + years) in the quota for the former, which might also explain why our sample average of age (that was elicited more precisely in years) is a little higher than the population average for the Netherlands.

9 In separate analyses, we examined the location of our respondents based on latitude and longitude coordinates. We investigated whether proximity (measured by the straight-line distance in kilometers between our respondents and the border of Zuid-Limburg) affects WTP for conservation by adding interaction terms between the tax increase attribute and distance. The interaction terms were included in more elaborate models than Model 3 and Model 4, i.e. for those who plan to visit Zuid-Limburg and for those who do not plan to visit Zuid-Limburg, respectively. The signs and significance levels of coefficient estimates attached to these interaction terms are positive and insignificant (coefficient estimates = 0.00001 and 0.00002; *p*-values = 0.105 and 0.126 for individuals who plan to visit Zuid-Limburg and those who do not plan to visit the area, respectively). Therefore, proximity to Zuid-Limburg is not significantly related to WTP for conservation among either subgroup.

10 The percentage of status quo choices is 26% overall.
