# Supplementary material for: The non-market value of reclaiming natural landscape and biodiversity: a Dutch case study
Source: Reg Environ Change. 2025 Jun 9;25(3):80. doi: 10.1007/s10113-025-02418-5 (PMC12149000; doi:10.1007/s10113-025-02418-5)
Supplement: Supplementary file 1 — Supplementary file1 (PDF 1991 KB) [file 10113_2025_2418_MOESM1_ESM.pdf]

Online Supplement: The non-market value of reclaiming natural landscape and biodiversity: A Dutch case study

Regional Environmental Change

Peter John Robinson[a], Marjolijn van Schendel[a], Jeroen C.J.H. Aerts[a], Wouter Botzen[a], Pieter van Beukering[a]

[a] Department of Environmental Economics, Institute for Environmental Studies, VU University Amsterdam, De Boelelaan 1087, 1081 HV Amsterdam, The Netherlands.

Corresponding author email: peter.robinson@vu.nl

Online Resource 1: Existing forest, grassland and wetland discrete choice experiment articles

Table A1: Existing forest, grassland and wetland discrete choice experiment articles subcategorized by country of investigation, ecosystem type and sample

Studies	Countries	Ecosystem type	Sample
Barkmann and Zschiegner (2010)	Germany	Grassland	Residents
Barrio and Loureiro (2013)	Spain	Forest	Residents
Berninger et al. (2010)	Canada, Finland	Forest	Environmentalists, forestry professionals, forest owners, indigenous populations, recreationalists
Bernues et al. (2014)	Spain	Forest, grassland	Residents
Birol et al. (2006)	Greece	Wetland	Residents
Birol and Cox (2007)	United Kingdom	Wetland	Residents
Brahic and Rambonilaza (2015)	France	Forest	Residents
Broadbent et al. (2010)	United States	Forest	Students
Cai et al. (2020)	China	Grassland	Herders
Carlsson et al. (2003)	Sweden	Forest, grassland, wetland	Residents
Cerda et al. (2014)	Chile	Forest	Residents
Colombo and Hanley (2008)	United Kingdom	Forest, grassland, wetland	Residents
Decker and Watson (2017)	United States	Grassland	Residents
de Ayala et al. (2015)	Spain	Forest	Residents
de Valck et al. (2014)	Belgium	Forest	Residents
Dias and Belcher (2015)	Canada	Wetland	Residents
Dissanayake and Ando (2014)	United States	Grassland	Residents
Do and Bennett (2009)	Vietnam	Wetland	Residents
Elsasser et al. (2010)	Germany	Forest, grassland	Residents

Farreras and Mavsar (2012)	Spain	Forest	Residents
Garrod et al. (2009)	United Kingdom	Forest	Residents
Giergiczny et al. (2015)	Poland	Forest	Visitors
Glenk and Martin-Ortega (2018)	United Kingdom	Wetland	Residents
Hanley et al. (1998)	United Kingdom	Forest, grassland	Residents, visitors
Hanley et al. (2007)	United Kingdom	Forest, grassland, wetland	Residents
Hasund et al. (2011)	Sweden	Grassland	Residents
Hoehn et al. (2010)	United States	Wetland	Residents
Horne et al. (2005)	Finland	Forest	Visitors
Hoyos et al. (2012)	Spain	Forest	Residents
Huber et al. (2011)	Switzerland	Forest	Politicians
Kefale et al. (2021)	Ethiopia	Forest	Farmers
Koetse et al. (2017)	Netherlands	Forest, grassland	Residents
Mallawaarachchi et al. (2001)	Australia	Forest, wetland	Residents
Mao et al. (2020)	China	Wetland	Residents
Meyerhoff et al. (2009)	Germany	Forest	Residents
Mombo et al. (2014)	Tanzania	Forest, wetland	Residents
Müller et al. (2020)	Switzerland	Forest	Residents, foresters
Naidoo and Adamowicz (2005)	Uganda	Forest	Visitors
Newell and Swallow (2013)	United States	Wetland	Residents
Nie et al. (2023)	China	Wetland	Residents
Nordén et al. (2017)	Sweden	Forest	Residents, forest owners, forest officials
Obeng et al. (2021)	Ghana	Forest	Residents
Olsen (2009)	Denmark	Forest, wetland	Residents
Pelletier et al. (2022)	Australia	Wetland	Residents

Rewitzer et al. (2017)	Switzerland	Forest, grassland	Residents
Schaafsma et al. (2014)	Belgium	Forest, grassland, wetland	Residents
Senzaki et al. (2017)	Japan	Wetland	Residents
Shi et al. (2021)	China	Grassland	Herders
Sinclair et al. (2021)	India	Wetland	Residents
Tan et al. (2018)	China	Wetland	Residents
Tu and Abildtrup (2016)	France	Forest	Residents
Upton et al. (2012)	Ireland	Forest	Residents
Valasiuk et al. (2018)	Belarus	Grassland, wetland	Residents
van Zanten et al. (2016)	Netherlands	Forest	Residents
Vecchiato and Tempesta (2013)	Italy	Forest, grassland	Residents
Weller and Elsasser (2018)	Germany	Forest	Residents
Westerberg et al. (2010)	France	Wetland	Residents
Xu and He (2022)	China	Wetland	Visitors

Notes:

The studies are restricted to those published in academic journals, therefore the large body of grey literature on the topic of landscape valuation has been omitted.

Online Resource 2: Instructional text for the discrete choice experiment

Instructies

Zoals op de kaart te zien is, zijn er op dit moment in Zuid-Limburg een aantal verschillende vormen van landgebruik.

Stedelijk gebied	20.408 hectares
Landbouw	34.796 hectares
Weiland	4.818 hectares
Bos	4.730 hectares
Natuurlijk grasland	333 hectares
Moeras	57 hectares

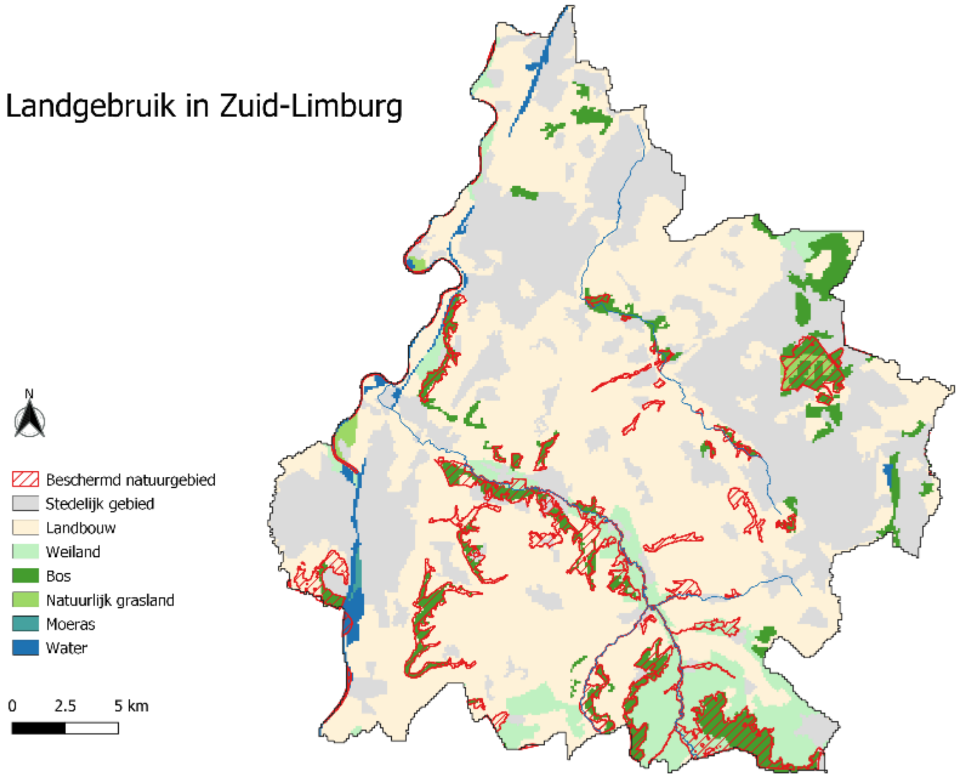

Volgende

English translation:

Instructions

As can be seen on the map, there are currently a number of different types of land use in Zuid-Limburg.

Urban area	*20,408 hectares*
Arable area	*34,796 hectares*

Pasture	*4,818 hectares*
Forest	*4,730 hectares*
Natural grassland	*333 hectares*
Wetland	*57 hectares*

Land use in Zuid-Limburg

Protected nature reserve

Urban area

Arable area

Pasture

Forest

Natural grassland

Wetland

Water

Fig. A1: First page of instructions

Bovendien leven er een aantal voor Zuid-Limburg bijzondere diersoorten die momenteel ofwel **kwetsbaar** zijn (bijv. IJsvogel, Geelgors, Wijngaardslak, Zeggekorfslak, Koninginnenpage, Vroedmeesterpad en Bunzing), of **bedreigd** (bijv. Grauwe klauwier, Grote gele kwikstaart, Vliegend hert, Vuursalamander, Hazelmuis en Grijze grootoorvleermuis) of **ernstig bedreigd** (bijv. Veldparelmoervlinder, Geelbuikvuurpad, Wilde hamster en Eikelmuis).

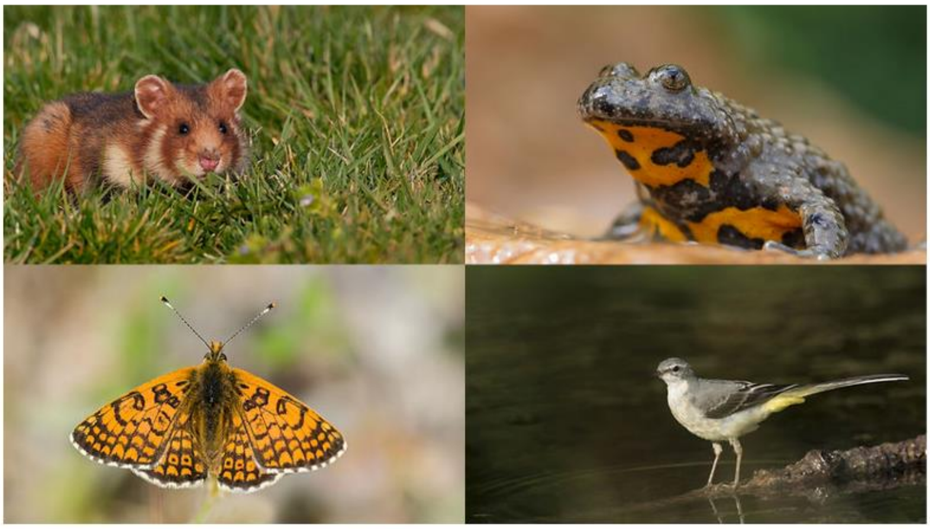

Volgende

English translation:

In addition, there are a number of animal species that are characteristic of Zuid-Limburg that are currently either vulnerable (e.g. Common kingfisher, Yellowhammer, Burgundy snail, Desmoulin's whorl snail, Old World swallowtail, Common midwife toad and European polecat), or endangered (e.g. Red-backed shrike, Western yellow wagtail, European stag beetle, Fire salamander, Hazel dormouse and Grey long-eared bat) or critically endangered (e.g. Glanville fritillary, Yellow-bellied toad, European hamster and Garden dormouse).

Fig. A2: Second page of instructions

Boeren kunnen bijdragen aan natuur en biodiversiteit via natuurinclusieve landbouwmethoden zoals grasvariëteit (kruidachtige en bloemrijke weiden), vermindering van het gebruik van pesticiden en meststoffen en herstel van natuurlijke landschapselementen rond boerderijen. Tevens zorgt rust in het maaibeheer ervoor dat vogels kunnen broeden en hun jongen kunnen grootbrengen.

Op dit moment draagt circa 50% van de landbouwbedrijven in Zuid-Limburg in meer of mindere mate bij aan natuurinclusieve landbouw.

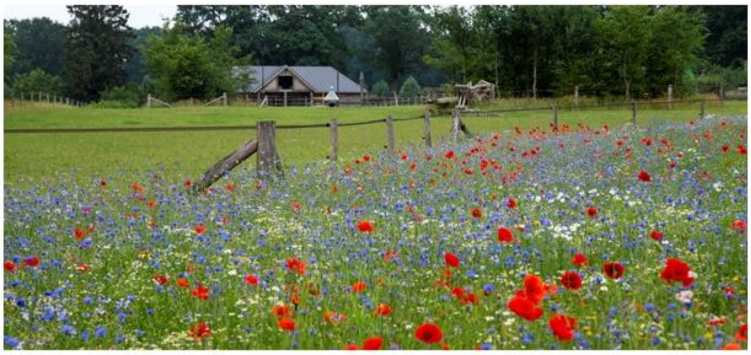

Volgende

English translation:

Farmers can contribute to nature and biodiversity through nature-inclusive farming methods such as grass variety (herbaceous and flowery meadows), reduction of pesticide and fertilizer use and restoration of natural landscape elements around farms. In addition, reducing noise whilst mowing ensures that birds can breed and raise their young.

Currently, approximately 50% of farming companies in Zuid-Limburg contribute to nature-inclusive agriculture.

Fig. A3: Third page of instructions

Er kunnen maatregelen worden genomen om de omvang van natuurgebieden, biodiversiteit en het aantal landbouwbedrijven dat natuurinclusieve landbouwmethoden toepast, te vergroten. Bijvoorbeeld via bescherming- en herstelactiviteiten, transformatie van bestaande gronden naar natuurgebieden en directe financiering voor boeren om natuurinclusieve landbouw te beoefenen. We zijn geïnteresseerd in uw voorkeuren voor deze wijzigingen.

In principe kunnen de wijzigingen worden ondersteund door hogere jaarlijkse gemeentelijke belastingen voor huishoudens, waarvan de inkomsten gebruikt worden om bovenstaande maatregelen te betalen.

Volgende

English translation:

Measures can be taken to increase the size of natural landscape areas, biodiversity and the number of farms applying nature-inclusive farming methods. For example, through protection and restoration activities, transformation of existing land into natural landscapes and direct funding for farmers to practice nature-inclusive agriculture. We are interested in your preferences for these changes.

In principle, the changes can be supported by higher annual municipal taxes for households, the revenues of which would be used to pay for the above measures.

Fig. A4: Fourth page of instructions

U wordt straks gevraagd om te kiezen tussen drie opties die iets zeggen over de staat van de natuur en het landschap in Zuid-Limburg. Elke optie bestaat uit een combinatie van een aantal kenmerken en een een prijsindicatie voor de verhoging van de gemeentebelasting per huishouden. De verschillende kenmerken zijn:

Aantal bedreigde diersoorten in Zuid-Limburg *(momenteel zijn er 17 kwetsbare, bedreigde of ernstig bedreigde diersoorten die speciaal zijn voor Zuid-Limburg)*:

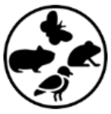

- geen verandering;
- 5 diersoorten worden niet meer bedreigd;
- 10 diersoorten worden niet meer bedreigd;
- 15 diersoorten worden niet meer bedreigd.

Oppervlakte bos *(momenteel is er 4.730 hectare bosgebied)*:

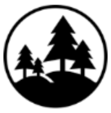

- geen verandering;
- +100 hectare (140 voetbalvelden meer);
- +200 hectare (280 voetbalvelden meer);
- +500 hectare (700 voetbalvelden meer).

Oppervlakte natuurlijk grasland *(momenteel is er 333 hectare natuurlijk grasland)*:

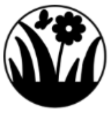

- geen verandering;
- +100 hectare (140 voetbalvelden meer);
- +200 hectare (280 voetbalvelden meer);
- +500 hectare (700 voetbalvelden meer).

Oppervlakte moeras *(momenteel is er 57 hectare moeras)*:

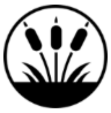

- geen verandering;
- +100 hectare (140 voetbalvelden meer);
- +200 hectare (280 voetbalvelden meer);
- +500 hectare (700 voetbalvelden meer).

Natuurinclusieve landbouw *(momenteel draagt ongeveer 50% van de landbouwbedrijven bij aan natuurinclusieve landbouw)*:

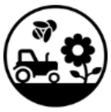

- geen verandering (50% van de landbouwbedrijven draagt bij aan natuurinclusieve landbouw);
- +30 procentpunt (80% van de landbouwbedrijven draagt dan bij aan natuurinclusieve landbouw);
- +50 procentpunt (100% van de landbouwbedrijven draagt dan bij aan natuurinclusieve landbouw).

Jaarlijkse verhoging gemeentebelasting in euro's (per huishouden), ofwel: geen verandering, ofwel een blijvende verhoging van € 20, € 60, € 120 of € 240 op uw jaarlijkse gemeentebelasting, waarvan de opbrengst geoormerkt is voor natuurmaatregelen.

Een stijging in oppervlak van het ene landgebruik heeft logischerwijs het effect dat een ander landgebruik in oppervlakte afneemt. Het land dat wordt opgeofferd om ofwel de oppervlakte van bos, natuurlijk grasland of moeras te vergroten, zal in gelijke hectaren worden afgenomen van stedelijk gebied, landbouwgrond en weilanden.

Volgende

English translation:

You will soon be asked to choose between three options that are characterized by the state of nature and the natural landscape in Zuid-Limburg. Each option consists of a combination of a number of characteristics and a price related to the increase in municipal tax per household. The different characteristics are:

Number of threatened species in Zuid-Limburg *(there are currently 17 vulnerable, endangered or critically endangered species that are characteristic of Zuid-Limburg):*

no change;

5 animal species are no longer threatened;

10 animal species are no longer threatened;

15 animal species are no longer threatened.

Forest area *(currently there are 4,730 hectares of forest area):*

no change;

+100 hectares (140 football fields more);

+200 hectares (280 more football fields);

+500 hectares (700 more football fields).

Natural grassland area *(currently there are 333 hectares of natural grassland):*

no change;

+100 hectares (140 football fields more);

+200 hectares (280 more football fields);

+500 hectares (700 more football fields).

Wetland area *(currently there are 57 hectares of wetland):*

no change;

+100 hectares (140 football fields more);

+200 hectares (280 more football fields);

+500 hectares (700 more football fields).

Nature-inclusive farming *(currently around 50% of agriculture business is contributing to some sort of nature-inclusive farming):*

no change (50% of agricultural companies contribute to nature-inclusive farming);

+30 percentage points (80% of agricultural companies then contribute to nature-inclusive farming);

+50 percentage points (100% of agricultural companies then contribute to nature-inclusive farming).

Annual increase in municipal tax in euros (per household)*, either: no change or a permanent increase of €20, €60, €120 or €240 on your annual municipal tax, the proceeds of which are earmarked for nature measures.*

An increase in the area of one land use logically has the effect of reducing the area of another land use. The land that is sacrificed to increase either the area of forest, natural grassland or wetland will be taken in equal hectares from urban areas, arable areas and pastures.

Fig. A5: Fifth page of instructions

Vervolgens wordt u gevraagd te kiezen tussen drie opties, bijvoorbeeld:

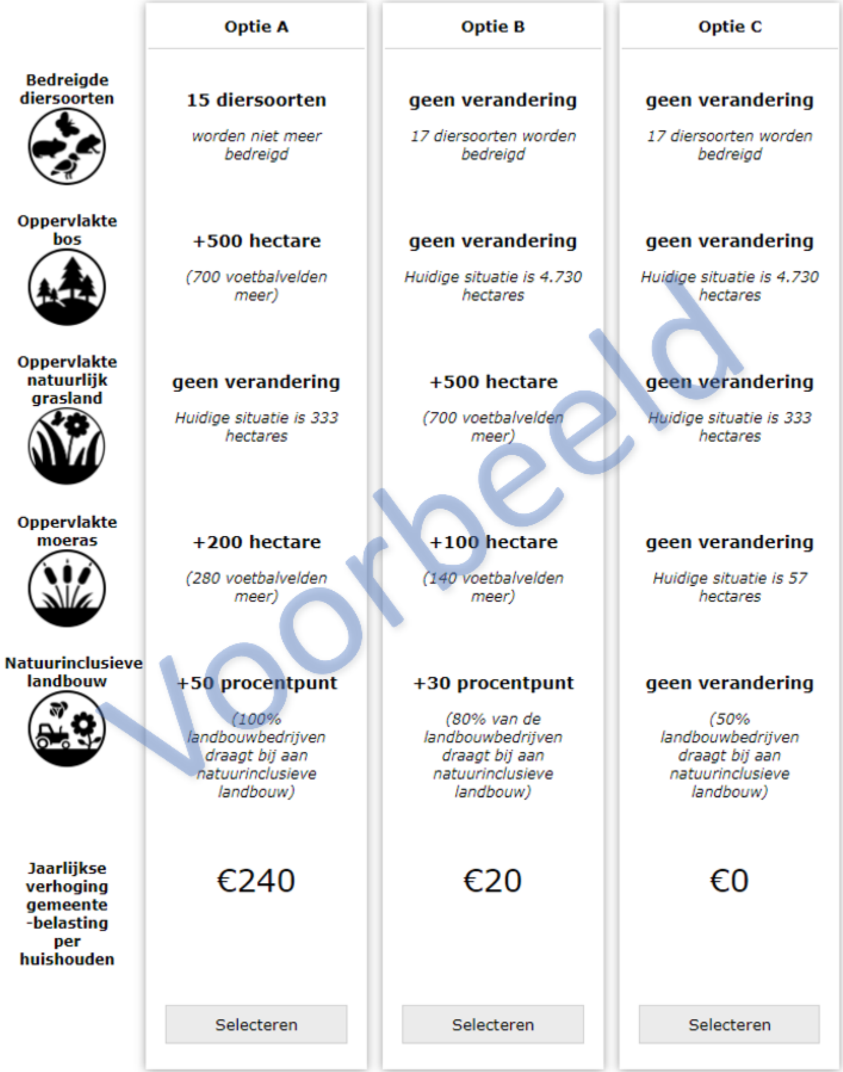

	Optie A	Optie B	Optie C
**Bedreigde diersoorten**	**15 diersoorten** *worden niet meer bedreigd*	**geen verandering** *17 diersoorten worden bedreigd*	**geen verandering** *17 diersoorten worden bedreigd*
**Oppervlakte bos**	**+500 hectare** *(700 voetbalvelden meer)*	**geen verandering** *Huidige situatie is 4.730 hectares*	**geen verandering** *Huidige situatie is 4.730 hectares*
**Oppervlakte natuurlijk grasland**	**geen verandering** *Huidige situatie is 333 hectares*	**+500 hectare** *(700 voetbalvelden meer)*	**geen verandering** *Huidige situatie is 333 hectares*
**Oppervlakte moeras**	**+200 hectare** *(280 voetbalvelden meer)*	**+100 hectare** *(140 voetbalvelden meer)*	**geen verandering** *Huidige situatie is 57 hectares*
**Natuurinclusieve landbouw**	**+50 procentpunt** *(100% landbouwbedrijven draagt bij aan natuurinclusieve landbouw)*	**+30 procentpunt** *(80% van de landbouwbedrijven draagt bij aan natuurinclusieve landbouw)*	**geen verandering** *(50% landbouwbedrijven draagt bij aan natuurinclusieve landbouw)*
**Jaarlijkse verhoging gemeente-belasting per huishouden**	€240	€20	€0
	Selecteren	Selecteren	Selecteren

Opties A en B vertegenwoordigen **twee verschillende scenario's op basis van extra maatregelen** die met uw jaarlijkse bijdrage worden gefinancierd.

Optie C toont het **'niks doen'-scenario** zonder extra maatregelen.

Kies steeds uit de drie opties met combinaties van kenmerken de optie die u het beste vindt en waarvoor u bereid bent om het aangegeven bedrag te betalen.

Volgende

English translation:

You will now be asked to choose between three options, for example:

{The watermark reads "example". Options are "Option A", "Option B" and "Option C". Attributes listed in the left-hand column read "Threatened animal species", "Forest area",

"Natural grassland area", "Wetland area", "Nature-inclusive farming" and "Annual increase in municipal tax (per household)". For the "Threatened animal species" attribute, the attribute levels list the number of species that "are no longer threatened" or "are threatened" in case of "no change". For the "Forest area" attribute, the attribute levels list the increase in hectares and how many "more football fields" this equates to or that the "current situation is 4,730 hectares" in case of "no change". The "Natural grassland area" and "Wetland area" attributes follow the same logic. The "Nature-inclusive farming" attribute levels list the percent "of agricultural companies" that "contribute to nature-inclusive farming" and the increase in "percentage points". The "Annual increase in municipal tax (per household)" attribute levels display the euro amount of the increase.}

Options A and B represent two different scenarios based on additional measures financed with your annual contribution.

Option C shows the 'do nothing' scenario with no additional measures.

Always choose from the three options with combinations of characteristics the option that you prefer most and for which you are willing to pay the indicated amount.

Fig. A6: Sixth page of instructions

Bedenk zorgvuldig hoeveel extra geld u zich elk jaar kunt veroorloven om bij te dragen aan het behoud van de Zuid-Limburgse natuur.

In totaal krijgt u 6 keuzekaarten te zien en wordt u gevraagd op elke kaart één optie te kiezen. **In het onwaarschijnlijke geval dat een van deze kaarten niet wordt getoond, vernieuw dan uw browser pagina**.

Volgende

English translation:

Consider carefully how much extra money you can afford to spend each year to contribute to the conservation of Zuid-Limburg's nature.

In total you will be presented with 6 choice cards and asked to choose one option on each card. ***In the unlikely event that one of these cards is not displayed, please refresh your browser page.***

Fig. A7: Seventh page of instructions

Online Resource 3: Conservation status of animal species characteristic of Zuid-Limburg

Table A2: Animal species ranked by conservation status

**Critically endangered**	**Endangered**	**Vulnerable**
Glanville fritillary *Melitaea cinxia*	Red-backed shrike *Lanius collurio*	Common kingfisher *Alcedo atthis*
Yellow-bellied toad *Bombina variegata*	Western yellow wagtail *Motacilla flava*	Yellowhammer *Emberiza citrinella*
European hamster *Cricetus cricetus*	European stag beetle *Lucanus cervus*	Burgundy snail *Helix pomatia*
Garden dormouse *Eliomys quercinus*	Fire salamander *Salamandra salamandra*	Desmoulin's whorl snail *Vertigo moulinsiana*
	Hazel dormouse *Muscardinus avellanarius*	Old World swallowtail *Papilio machaon*
	Grey long-eared bat *Plecotus austriacus*	Common midwife toad *Alytes obstetricans*
		European polecat *Mustela putorius*

Online Resource 4: Example choice set presented to respondents

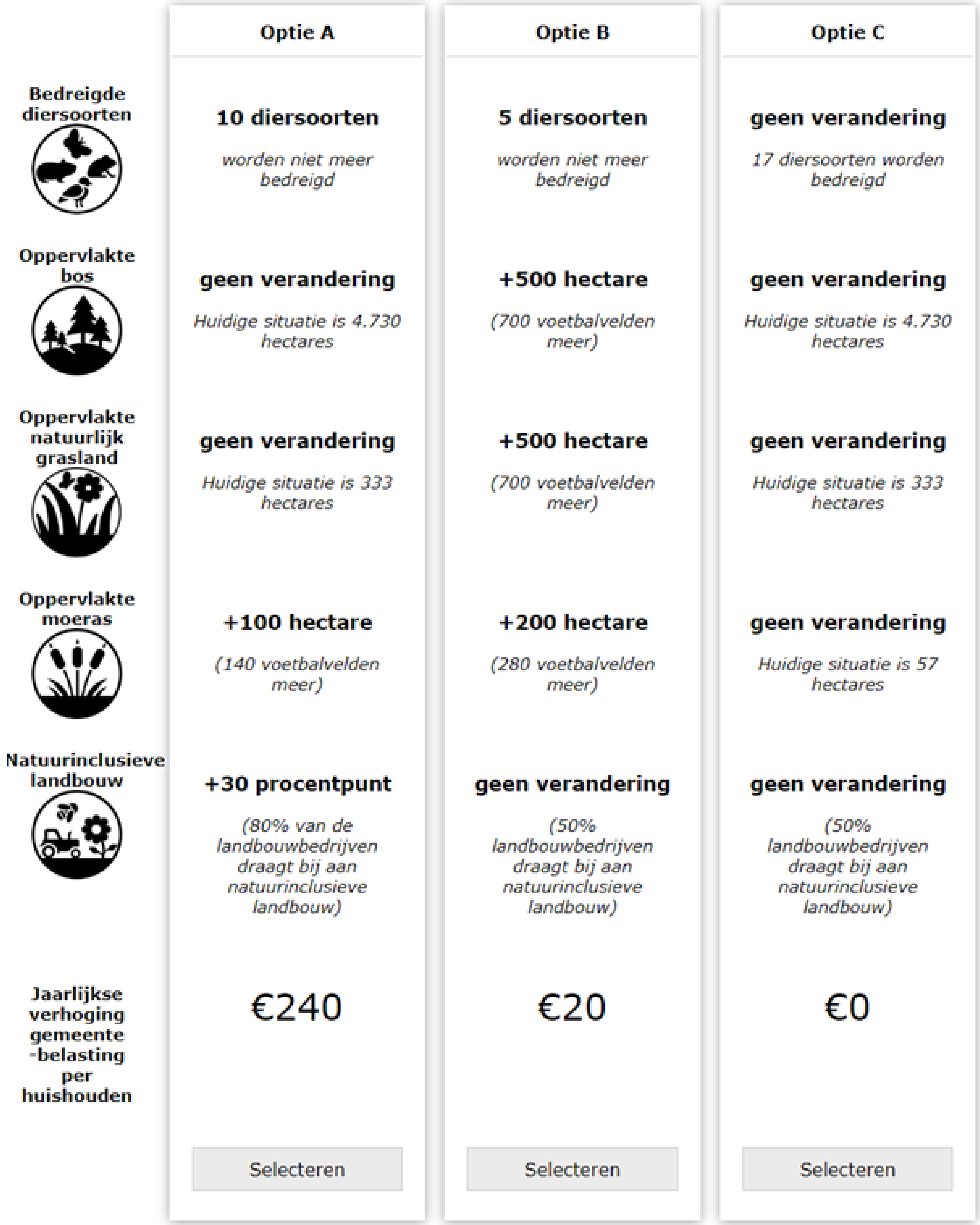

Fig. A8: Example choice set

Notes: Options are “Option A”, “Option B” and “Option C”. Attributes listed in the left-hand column read “Threatened animal species”, “Forest area”, “Natural grassland area”, “Wetland area”, “Nature-inclusive farming” and “Annual increase in municipal tax (per household)”. For the “Threatened animal species” attribute, the attribute levels list the number of species that “are no longer threatened” or “are threatened” in case of “no change”. For the “Forest area” attribute, the attribute levels list the increase in hectares and how many “more football fields” this equates to or that the “current situation is 4,730 hectares” in case of “no change”. The “Natural grassland area” and “Wetland area” attributes follow the same logic. The “Nature-inclusive farming” attribute levels list the percent “of agricultural companies” that “contribute to nature-inclusive farming” and the increase in “percentage points”. The “Annual increase in municipal tax (per household)” attribute levels display the euro amount of the increase.

Online Resource 5: Descriptive statistics and elicitation and coding of variables

Table A3: Descriptive statistics			
**Variable**	**Values**	**Elicitation**	**Coding**
Age	48.27 (17.90)<br>N = 1,288	How old are you, in years (if you prefer not to say then leave blank)?	Age in years; missing = prefer not to say
Male	0.47<br>N = 1,294	What is your gender?	1 = male; 0 = female; missing = other or prefer not to say
Higher education	0.31<br>N = 1,283	What is your highest completed level of education?	1 = university/college graduate or higher; 0 = several years of secondary education, completed secondary education or several years of university/college; missing = other or prefer not to say
Income	1 = 3% of respondents; 2 = 20% of respondents; 3 = 41% of respondents; 4 = 23% of respondents; 5 = 11% of respondents; 6 = 2% of respondents<br>N = 1,063	Which of the following ranges best describes your monthly household income for 2022 before taxes?	1 = less than €800; 2 = €800 to €1,999; 3 = €2,000 to €3,999; 4 = €4,000 to €5,900; 5 = €6,000 to €9,999; 6 = more than €10,000; missing = don't know or prefer not to say
Visited Zuid-Limburg in the past ten years	0.91<br>N = 1,297	Have you visited Zuid-Limburg before?	1 = yes; 0 = no
Planning to visit Zuid-Limburg in the future	0.65<br>N = 1,297	Are you planning to visit Zuid-Limburg in the future?	1 = yes; 0 = no
Concern extinction	1 = 6% of respondents; 2 = 16% of respondents; 3 = 29% of respondents; 4 = 29% of respondents; 5 =	In your everyday life, how concerned do you feel about the following issues? (The extinction of animal and/or plant species)	1 = I never think about it; 2 = I think about it but I'm not at all concerned; 3 = I'm a little bit concerned; 4 = I'm moderately concerned; 5 = I'm seriously concerned

	20% of respondents<br><br>N = 1,297		
Concern built-up area	1 = 8% of respondents; 2 = 17% of respondents; 3 = 29% of respondents; 4 = 29% of respondents; 5 = 17% of respondents<br><br>N = 1,297	In your everyday life, how concerned do you feel about the following issues? (Increase in built-up area)	1 = I never think about it; 2 = I think about it but I'm not at all concerned; 3 = I'm a little bit concerned; 4 = I'm moderately concerned; 5 = I'm seriously concerned
Concern nitrogen crisis	1 = 9% of respondents; 2 = 23% of respondents; 3 = 28% of respondents; 4 = 25% of respondents; 5 = 14% of respondents<br><br>N = 1,297	In your everyday life, how concerned do you feel about the following issues? (The 'nitrogen crisis')	1 = I never think about it; 2 = I think about it but I'm not at all concerned; 3 = I'm a little bit concerned; 4 = I'm moderately concerned; 5 = I'm seriously concerned
Patience	60.34 (21.34)<br><br>N = 1,297	How willing are you to give up something that is beneficial for you today in order to benefit more from that in the future?	0 = completely unwilling to do so; …; 100 = very willing to do so
Notes: The mean or proportion is provided with the standard deviation in parentheses.			

Online Resource 6: Welfare measures

Table A4: Mean willingness-to-pay (WTP) (Euros) for changes to attributes

	**Pooled**	**Plan to visit**	**Plan to visit and visited before for outdoor recreation**	**Plan to visit and did not visit before for outdoor recreation**	**Plan to visit and high income**	**Plan to visit and low income**	**Do not plan to visit**	**Do not plan to visit and visited before for outdoor recreation**	**Do not plan to visit and did not visit before for outdoor recreation**	**Do not plan to visit and high income**	**Do not plan to visit and low income**
Number species off threatened status	5.651*** (0.398)	6.344*** (0.551)	7.716*** (0.822)	4.929*** (0.750)	7.580*** (1.054)	6.134*** (0.843)	4.550*** (0.555)	3.661*** (0.919)	4.835*** (0.701)	4.483*** (1.139)	4.328*** (0.708)
Forest coverage (one hundred hectares)	3.337*** (1.167)	3.335** (1.559)	3.421 (2.285)	2.740 (2.136)	4.568* (2.537)	3.072 (2.544)	3.382** (1.697)	6.296* (3.258)	2.409 (1.985)	2.958 (4.034)	3.671* (2.126)
Natural grassland coverage (one hundred hectares)	2.332* (1.227)	1.568 (1.654)	2.275 (2.420)	1.264 (2.310)	3.663 (3.109)	-0.856 (2.450)	3.966** (1.782)	5.577* (2.950)	3.146 (2.215)	6.353* (3.649)	3.301 (2.441)
Wetland coverage (one hundred hectares)	3.933*** (1.229)	3.918** (1.652)	4.333* (2.352)	3.479 (2.363)	1.383 (2.985)	5.213** (2.370)	3.402* (1.851)	5.855* (3.254)	2.462 (2.333)	2.655 (4.277)	3.212 (2.428)
Nature-inclusive farming (percentage points)	0.173 (0.200)	0.252 (0.277)	0.376 (0.392)	0.087 (0.389)	0.769 (0.470)	0.250 (0.390)	0.082 (0.268)	0.730 (0.462)	-0.144 (0.325)	1.152* (0.620)	-0.126 (0.332)

Notes: ***, **, *: Significance at 1%, 5%, 10% level, respectively based on the Krinsky and Robb (1986) bootstrapping method with 1,000 random draws. Standard errors are in parentheses.

Online Resource 7: Full survey text

<u>Page 1 (if respondent selects 2nd option end survey)</u>

Dit onderzoek gaat over de economische waarde die mensen geven aan het Zuid-Limburgse landschap en natuur. Het onderzoek wordt uitgevoerd door het Instituut voor Milieuvraagstukken (IVM) van de Vrije Universiteit Amsterdam (VU). U moet minstens 18 jaar oud zijn om deel te nemen. Het invullen van de enquête neemt ongeveer 10 tot 15 minuten in beslag. Uw deelname is vrijwillig, wat betekent dat u op elk moment kunt afhaken. Uw privacy wordt beschermd voor zover dat wettelijk is toegestaan. Er zal geen persoonlijk identificeerbare informatie worden opgenomen in enig onderzoeksproduct. Alle gegevens van het onderzoek zullen worden opgeslagen op een veilige, met een wachtwoord beveiligde locatie, en zullen binnen tien jaar na het begin van het onderzoek worden vernietigd. Als u vragen of opmerkingen heeft, kunt u contact opnemen met: peter.robinson@vu.nl. Als u instemt met deelname aan dit onderzoek, betekent dit dat u de bovenstaande informatie heeft gelezen en begrepen.

Gaat u akkoord met deelname aan dit onderzoek?

◯ Ja, ik ga akkoord met deelname ◯ Nee, ik ga niet akkoord met deelname

Volgende

English translation:

This research is about the economic value that people attach to the Zuid-Limburg landscape and nature. The research is conducted by the Institute for Environmental Studies (IVM) of the Vrije Universiteit Amsterdam (VU). You must be at least 18 years old to participate. Completing the survey will take approximately 10 to 15 minutes. Your participation is voluntary, which means that you can withdraw at any time. Your privacy will be protected to the extent permitted by law. No personally identifiable information will be included in any research product. All data from the research will be stored in a secure, password-protected location and will be destroyed within ten years of the start of the research. If you have any questions or comments, please contact: peter.robinson@vu.nl. By agreeing to participate in this research, you signify that you have read and understood the above information.

Do you agree to participate in this study?

-Yes, I agree to participate

-No, I do not agree to participate

<u>Page 2</u>

Hoe oud bent u, in jaren (als u niets wilt zeggen, laat het dan open)?

[ ] jaar

Volgende

English translation:

How old are you, in years (if you prefer not to say then leave blank)?

…years

Page 3

Wat is uw geslacht?

- ◯ Man
- ◯ Vrouw
- ◯ Anders
- ◯ Zeg ik liever niet

Volgende

English translation:

What is your gender?

-Male

-Female

-Other

-Prefer not to say

Page 4

Welk van de volgende intervallen beschrijft het best uw maandelijkse huishoudinkomen voor 2022 vóór belastingen?

- ◯ Minder dan € 800
- ◯ € 800 tot € 1.999
- ◯ € 2.000 tot € 3.999
- ◯ € 4.000 tot € 5.999
- ◯ € 6.000 tot € 9.999
- ◯ € 10.000 of meer
- ◯ Weet ik niet
- ◯ Zeg ik liever niet

Volgende

English translation:

Which of the following options best describes your monthly household income for 2022 before taxes?

-Less than €800

-€800 to €1,999

-€2,000 to €3,999

-€4,000 to €5,999

-€6,000 to €9,999

-€10,000 or more

-I don't know

-Prefer not to say

Page 5

Wat is uw hoogst voltooide opleidingsniveau?

- Enkele jaren voortgezet onderwijs
- Afgerond voortgezet onderwijs
- Enkele jaren middelbaar beroepsonderwijs (mbo)
- Afgerond middelbaar beroepsonderwijs (mbo)
- Enkele jaren universiteit/hogeschool (wo/hbo)
- Afgestudeerd aan universiteit/hogeschool (wo/hbo)
- Postdoctoraal
- Anders, graag toelichten:
- Zeg ik liever niet

Volgende

English translation:

What is your highest completed level of education?

-Several years of secondary education

-Completed secondary education

-Several years of secondary vocational education (MBO)

-Completed secondary vocational education (MBO)

-Several years of university/college (WO/HBO)

-Graduated from university/college (WO/HBO)

-Postdoctoral

-Other, please explain:…

-Prefer not to say

Page 6

Welke van de volgende categorieën karakteriseert u het best?

- ◯ Voltijds werknemer
- ◯ Deeltijdwerknemer
- ◯ Werkgever
- ◯ Zelfstandige / Freelancer
- ◯ Ondernemer
- ◯ Werkloos / op zoek naar een baan
- ◯ Gepensioneerde
- ◯ Student
- ◯ Arbeidsongeschikt
- ◯ Anders, graag toelichten: [ ]
- ◯ Zeg ik liever niet

Volgende

English translation:

Which of the following categories best characterizes you?

-Full-time employee

-Part-time employee

-Employer

-Self-employed / Freelancer

-Entrepreneur

-Unemployed / looking for a job

-Retired

-Student

-Disabled

-Other, please explain:…

-I'd rather not say

Page 7

In welke gemeente woont u? [ ]

Volgende

English translation:

In which municipality do you live?

Page 8

De volgende vragen gaan over uw bekendheid en persoonlijke ervaringen met Zuid-Limburg.

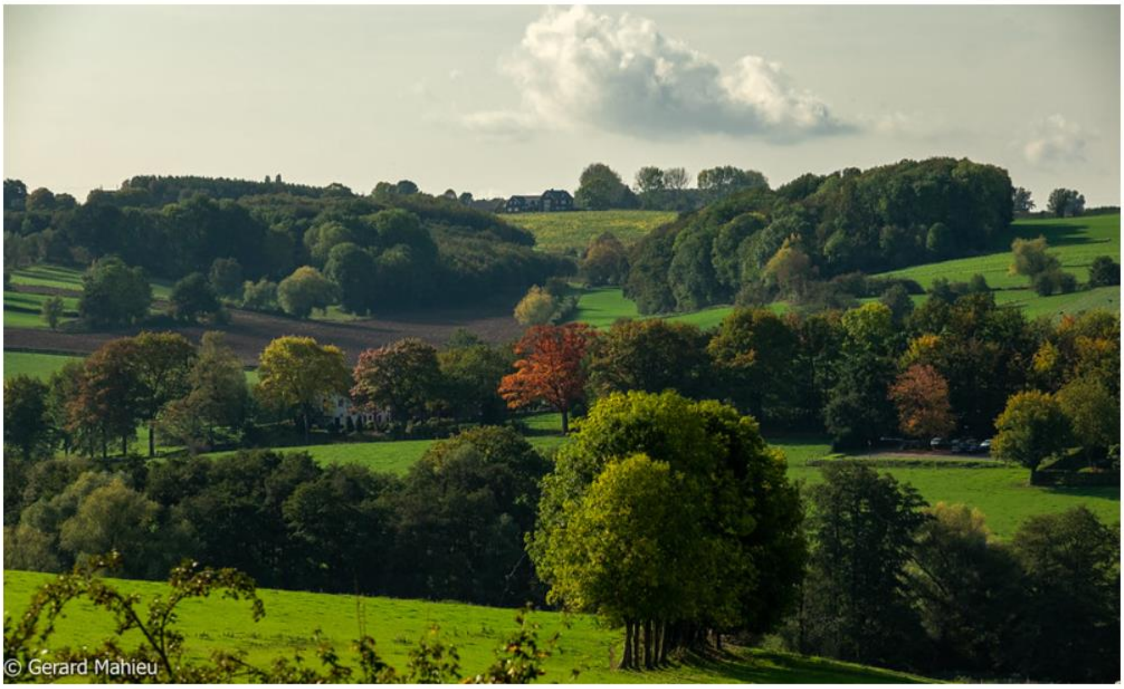

Volgende

English translation:

The following questions are about your familiarity with and personal experiences with Zuid-Limburg.

Page 9 (if respondent selects second option skip to page 12)

Heeft u Zuid-Limburg ooit eerder bezocht?

- Ja
- Nee

Volgende

English translation:

Have you ever visited Zuid-Limburg before?

-Yes

-No

Page 10

Geef bij benadering aan hoe vaak u in de afgelopen tien jaar Zuid-Limburg heeft bezocht.

- 1 keer
- 2 of 3 keer
- 4 of 5 keer
- 6 tot 10 keer
- 11 tot 15 keer
- Meer dan 15 keer
- Ik weet het niet

Volgende

English translation:

Please indicate approximately how often you have visited Zuid-Limburg in the past ten years.

-1 time

-2 or 3 times

-4 or 5 times

-6 to 10 times

-11 to 15 times

-More than 15 times

-Don't know

Page 11

Wat was uw reden voor bezoek aan Zuid-Limburg? *(Meerdere antwoorden mogelijk)*

- [ ] Stedentrip
- [ ] Werk
- [ ] Familiebezoek
- [ ] Outdoor avontuur
- [ ] Wandelen
- [ ] Fietsen
- [ ] Evenement
- [ ] Wellness
- [ ] Bezienswaardigheid (kastelen, kloosters, grotten, e.d.)
- [ ] Recreatief winkelen
- [ ] Anders ______

Volgende

English translation:

What was your reason for visiting Zuid-Limburg? (Multiple answers possible)

-City trip

-Work

-Family visit

-Outdoor adventure

-Walking

-Cycling

-Event

-Wellness

-Places of interest (castles, monasteries, caves, etc.)

-Recreational shopping

-Other: ...

Page 12

Bent u van plan om de komende tijd Zuid-Limburg te bezoeken?

- ◯ Ja
- ◯ Nee

Volgende

English translation:

Are you planning to visit Zuid-Limburg in the future?

-Yes

-No

Page 13

Instructies

Zoals op de kaart te zien is, zijn er op dit moment in Zuid-Limburg een aantal verschillende vormen van landgebruik.

Stedelijk gebied	20.408 hectares
Landbouw	34.796 hectares
Weiland	4.818 hectares
Bos	4.730 hectares
Natuurlijk grasland	333 hectares
Moeras	57 hectares

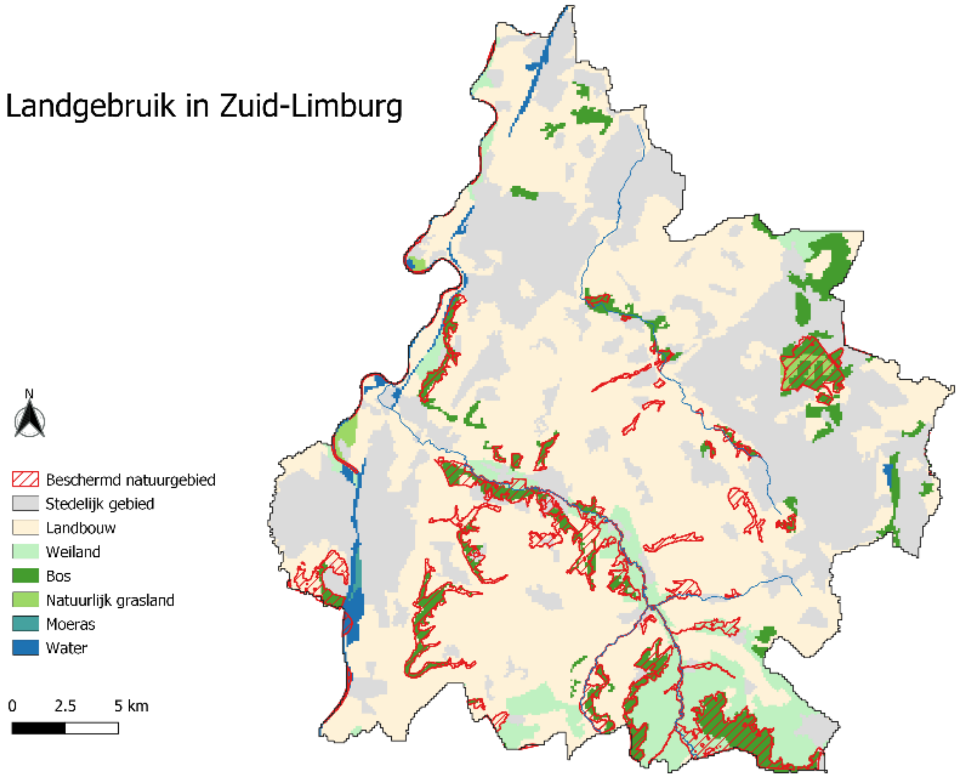

Volgende

English translation:

Instructions

As can be seen on the map, there are currently a number of different types of land use in Zuid-Limburg.

Urban area	*20,408 hectares*
Arable area	*34,796 hectares*
Pasture	*4,818 hectares*

Forest	*4,730 hectares*
Natural grassland	*333 hectares*
Wetland	*57 hectares*

Land use in Zuid-Limburg

Protected nature reserve

Urban area

Arable area

Pasture

Forest

Natural grassland

Wetland

Water

Page 14

Bovendien leven er een aantal voor Zuid-Limburg bijzondere diersoorten die momenteel ofwel **kwetsbaar** zijn (bijv. IJsvogel, Geelgors, Wijngaardslak, Zeggekorfslak, Koninginnenpage, Vroedmeesterpad en Bunzing), of **bedreigd** (bijv. Grauwe klauwier, Grote gele kwikstaart, Vliegend hert, Vuursalamander, Hazelmuis en Grijze grootoorvleermuis) of **ernstig bedreigd** (bijv. Veldparelmoervlinder, Geelbuikvuurpad, Wilde hamster en Eikelmuis).

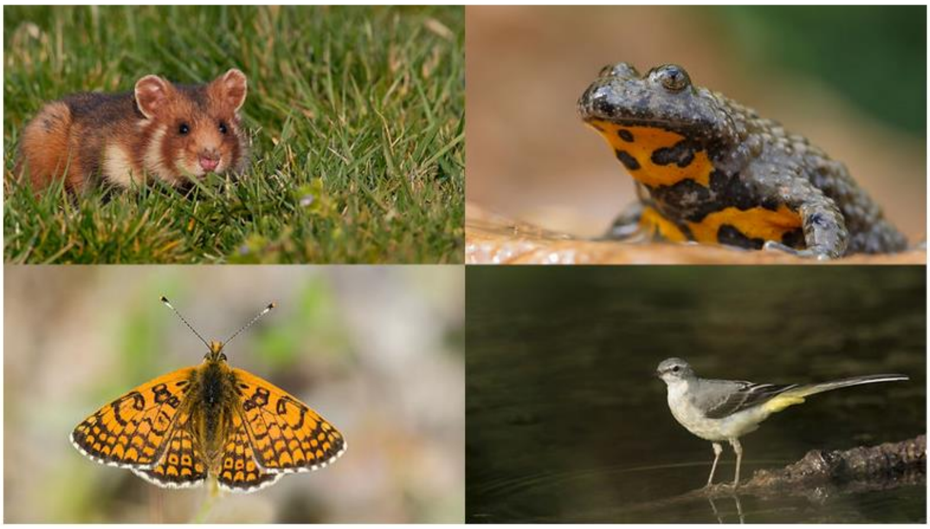

Volgende

English translation:

In addition, there are a number of animal species that are characteristic of Zuid-Limburg that are currently either vulnerable (e.g. Common kingfisher, Yellowhammer, Burgundy snail, Desmoulin’s whorl snail, Old World swallowtail, Common midwife toad and European polecat), or endangered (e.g. Red-backed shrike, Western yellow wagtail, European stag beetle, Fire salamander, Hazel dormouse and Grey long-eared bat) or critically endangered (e.g. Glanville fritillary, Yellow-bellied toad, European hamster and Garden dormouse).

Page 15

Boeren kunnen bijdragen aan natuur en biodiversiteit via natuurinclusieve landbouwmethoden zoals grasvariëteit (kruidachtige en bloemrijke weiden), vermindering van het gebruik van pesticiden en meststoffen en herstel van natuurlijke landschapselementen rond boerderijen. Tevens zorgt rust in het maaibeheer ervoor dat vogels kunnen broeden en hun jongen kunnen grootbrengen.

Op dit moment draagt circa 50% van de landbouwbedrijven in Zuid-Limburg in meer of mindere mate bij aan natuurinclusieve landbouw.

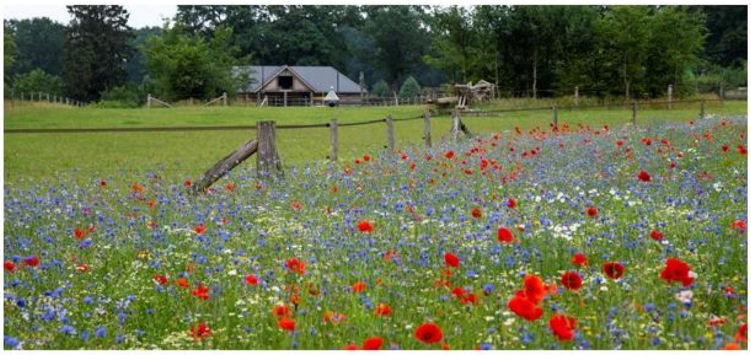

Volgende

English translation:

Farmers can contribute to nature and biodiversity through nature-inclusive farming methods such as grass variety (herbaceous and flowery meadows), reduction of pesticide and fertilizer use and restoration of natural landscape elements around farms. In addition, reducing noise whilst mowing ensures that birds can breed and raise their young.

Currently, approximately 50% of farming companies in Zuid-Limburg contribute to nature-inclusive agriculture.

Page 16

Er kunnen maatregelen worden genomen om de omvang van natuurgebieden, biodiversiteit en het aantal landbouwbedrijven dat natuurinclusieve landbouwmethoden toepast, te vergroten. Bijvoorbeeld via bescherming- en herstelactiviteiten, transformatie van bestaande gronden naar natuurgebieden en directe financiering voor boeren om natuurinclusieve landbouw te beoefenen. We zijn geïnteresseerd in uw voorkeuren voor deze wijzigingen.

In principe kunnen de wijzigingen worden ondersteund door hogere jaarlijkse gemeentelijke belastingen voor huishoudens, waarvan de inkomsten gebruikt worden om bovenstaande maatregelen te betalen.

Volgende

English translation:

Measures can be taken to increase the size of natural landscape areas, biodiversity and the number of farms applying nature-inclusive farming methods. For example, through protection and restoration

activities, transformation of existing land into natural landscapes and direct funding for farmers to practice nature-inclusive agriculture. We are interested in your preferences for these changes.

In principle, the changes can be supported by higher annual municipal taxes for households, the revenues of which would be used to pay for the above measures.

Page 17

U wordt straks gevraagd om te kiezen tussen drie opties die iets zeggen over de staat van de natuur en het landschap in Zuid-Limburg. Elke optie bestaat uit een combinatie van een aantal kenmerken en een een prijsindicatie voor de verhoging van de gemeentebelasting per huishouden. De verschillende kenmerken zijn:

Aantal bedreigde diersoorten in Zuid-Limburg *(momenteel zijn er 17 kwetsbare, bedreigde of ernstig bedreigde diersoorten die speciaal zijn voor Zuid-Limburg)*:

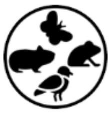

- geen verandering;
- 5 diersoorten worden niet meer bedreigd;
- 10 diersoorten worden niet meer bedreigd;
- 15 diersoorten worden niet meer bedreigd.

Oppervlakte bos *(momenteel is er 4.730 hectare bosgebied)*:

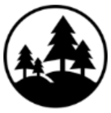

- geen verandering;
- +100 hectare (140 voetbalvelden meer);
- +200 hectare (280 voetbalvelden meer);
- +500 hectare (700 voetbalvelden meer).

Oppervlakte natuurlijk grasland *(momenteel is er 333 hectare natuurlijk grasland)*:

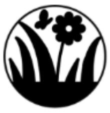

- geen verandering;
- +100 hectare (140 voetbalvelden meer);
- +200 hectare (280 voetbalvelden meer);
- +500 hectare (700 voetbalvelden meer).

Oppervlakte moeras *(momenteel is er 57 hectare moeras)*:

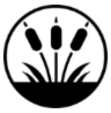

- geen verandering;
- +100 hectare (140 voetbalvelden meer);
- +200 hectare (280 voetbalvelden meer);
- +500 hectare (700 voetbalvelden meer).

Natuurinclusieve landbouw *(momenteel draagt ongeveer 50% van de landbouwbedrijven bij aan natuurinclusieve landbouw)*:

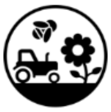

- geen verandering (50% van de landbouwbedrijven draagt bij aan natuurinclusieve landbouw);
- +30 procentpunt (80% van de landbouwbedrijven draagt dan bij aan natuurinclusieve landbouw);
- +50 procentpunt (100% van de landbouwbedrijven draagt dan bij aan natuurinclusieve landbouw).

Jaarlijkse verhoging gemeentebelasting in euro's (per huishouden), ofwel: geen verandering, ofwel een blijvende verhoging van € 20, € 60, € 120 of € 240 op uw jaarlijkse gemeentebelasting, waarvan de opbrengst geoormerkt is voor natuurmaatregelen.

Een stijging in oppervlak van het ene landgebruik heeft logischerwijs het effect dat een ander landgebruik in oppervlakte afneemt. Het land dat wordt opgeofferd om ofwel de oppervlakte van bos, natuurlijk grasland of moeras te vergroten, zal in gelijke hectaren worden afgenomen van stedelijk gebied, landbouwgrond en weilanden.

Volgende

English translation:

You will soon be asked to choose between three options that are characterized by the state of nature and the natural landscape in Zuid-Limburg. Each option consists of a combination of a number of characteristics and a price related to the increase in municipal tax per household. The different characteristics are:

Number of threatened species in Zuid-Limburg *(there are currently 17 vulnerable, endangered or critically endangered species that are characteristic of Zuid-Limburg):*

no change;

5 animal species are no longer threatened;

10 animal species are no longer threatened;

15 animal species are no longer threatened.

Forest area *(currently there are 4,730 hectares of forest area):*

no change;

+100 hectares (140 football fields more);

+200 hectares (280 more football fields);

+500 hectares (700 more football fields).

Natural grassland area *(currently there are 333 hectares of natural grassland):*

no change;

+100 hectares (140 football fields more);

+200 hectares (280 more football fields);

+500 hectares (700 more football fields).

Wetland area *(currently there are 57 hectares of wetland):*

no change;

+100 hectares (140 football fields more);

+200 hectares (280 more football fields);

+500 hectares (700 more football fields).

***Nature-inclusive farming** (currently around 50% of agriculture business is contributing to some sort of nature-inclusive farming):*

no change (50% of agricultural companies contribute to nature-inclusive farming);

+30 percentage points (80% of agricultural companies then contribute to nature-inclusive farming);

+50 percentage points (100% of agricultural companies then contribute to nature-inclusive farming).

***Annual increase in municipal tax in euros (per household)**, either: no change or a permanent increase of €20, €60, €120 or €240 on your annual municipal tax, the proceeds of which are earmarked for nature measures.*

An increase in the area of one land use logically has the effect of reducing the area of another land use. The land that is sacrificed to increase either the area of forest, natural grassland or wetland will be taken in equal hectares from urban areas, arable areas and pastures.

Page 18

Vervolgens wordt u gevraagd te kiezen tussen drie opties, bijvoorbeeld:

Voorbeeld

	Optie A	Optie B	Optie C
Bedreigde diersoorten	**15 diersoorten** *worden niet meer bedreigd*	**geen verandering** *17 diersoorten worden bedreigd*	**geen verandering** *17 diersoorten worden bedreigd*
Oppervlakte bos	**+500 hectare** *(700 voetbalvelden meer)*	**geen verandering** *Huidige situatie is 4.730 hectares*	**geen verandering** *Huidige situatie is 4.730 hectares*
Oppervlakte natuurlijk grasland	**geen verandering** *Huidige situatie is 333 hectares*	**+500 hectare** *(700 voetbalvelden meer)*	**geen verandering** *Huidige situatie is 333 hectares*
Oppervlakte moeras	**+200 hectare** *(280 voetbalvelden meer)*	**+100 hectare** *(140 voetbalvelden meer)*	**geen verandering** *Huidige situatie is 57 hectares*
Natuurinclusieve landbouw	**+50 procentpunt** *(100% landbouwbedrijven draagt bij aan natuurinclusieve landbouw)*	**+30 procentpunt** *(80% van de landbouwbedrijven draagt bij aan natuurinclusieve landbouw)*	**geen verandering** *(50% landbouwbedrijven draagt bij aan natuurinclusieve landbouw)*
Jaarlijkse verhoging gemeente-belasting per huishouden	€240	€20	€0
	Selecteren	Selecteren	Selecteren

Opties A en B vertegenwoordigen **twee verschillende scenario's op basis van extra maatregelen** die met uw jaarlijkse bijdrage worden gefinancierd.

Optie C toont het **'niks doen'-scenario** zonder extra maatregelen.

Kies steeds uit de drie opties met combinaties van kenmerken de optie die u het beste vindt en waarvoor u bereid bent om het aangegeven bedrag te betalen.

Volgende

English translation:

You will now be asked to choose between three options, for example:

{The watermark reads "example". Options are "Option A", "Option B" and "Option C". Attributes listed in the left-hand column read "Threatened animal species", "Forest area", "Natural grassland

area", "Wetland area", "Nature-inclusive farming" and "Annual increase in municipal tax (per household)". For the "Threatened animal species" attribute, the attribute levels list the number of species that "are no longer threatened" or "are threatened" in case of "no change". For the "Forest area" attribute, the attribute levels list the increase in hectares and how many "more football fields" this equates to or that the "current situation is 4,730 hectares" in case of "no change". The "Natural grassland area" and "Wetland area" attributes follow the same logic. The "Nature-inclusive farming" attribute levels list the percent "of agricultural companies" that "contribute to nature-inclusive farming" and the increase in "percentage points". The "Annual increase in municipal tax (per household)" attribute levels display the euro amount of the increase.}

Options A and B represent two different scenarios based on additional measures financed with your annual contribution.

Option C shows the 'do nothing' scenario with no additional measures.

Always choose from the three options with combinations of characteristics the option that you prefer most and for which you are willing to pay the indicated amount.

<u>Page 19</u>

Bedenk zorgvuldig hoeveel extra geld u zich elk jaar kunt veroorloven om bij te dragen aan het behoud van de Zuid-Limburgse natuur.

In totaal krijgt u 6 keuzekaarten te zien en wordt u gevraagd op elke kaart één optie te kiezen. **In het onwaarschijnlijke geval dat een van deze kaarten niet wordt getoond, vernieuw dan uw browser pagina**.

Volgende

English translation:

Consider carefully how much extra money you can afford to spend each year to contribute to the conservation of Zuid-Limburg's nature.

In total you will be presented with 6 choice cards and asked to choose one option on each card. ***In the unlikely event that one of these cards is not displayed, please refresh your browser page.***

Pages 20, 21, 22, 23, 24 and 25 are discrete choice experiment tasks

Kies EEN van de drie opties:
(1 van 6)

{Insert choice set}

Volgende

English translation:

Choose ONE of the three options:

(1 of 6)

Page 26

Hoe heeft u uw keuzes gemaakt?

- ◯ Alle zes de kenmerken tegelijk in overweging genomen
- ◯ Vijf van de kenmerken in overweging genomen
- ◯ Vier van de kenmerken in overweging genomen
- ◯ Drie van de kenmerken in overweging genomen
- ◯ Twee van de kenmerken in overweging genomen
- ◯ Slechts één van de kenmerken in overweging genomen
- ◯ Ik heb mijn intuïtie gebruikt
- ◯ Willekeurige keuzes gemaakt
- ◯ Ik weet het niet
- ◯ Anders, graag toelichten: ____

Volgende

English translation:

How did you make your choices?

-All six characteristics considered simultaneously

-Five of the characteristics considered

-Four of the characteristics considered

-Three of the characteristics considered

-Two of the characteristics considered

-Only one of the characteristics considered

-I used my intuition

-Made random choices

-Don't know

-Other, please explain:…

Page 27

Bij het maken van uw keuze, hoe belangrijk waren de volgende kenmerken voor u?

	Zeer onbelangrijk	Onbelangrijk	Neutraal	Belangrijk	Zeer belangrijk
Aantal diersoorten dat niet meer bedreigd wordt in Zuid-Limburg	○	○	○	○	○
Oppervlakte bos	○	○	○	○	○
Oppervlakte natuurlijk grasland	○	○	○	○	○
Oppervlakte moeras	○	○	○	○	○
Natuurinclusieve landbouw	○	○	○	○	○
Jaarlijkse verhoging gemeentebelasting	○	○	○	○	○

Volgende

English translation:

When making your choice, how important were the following characteristics to you?

{The attributes are listed in the first column with options listed as follows: "Very unimportant", "Unimportant", "Neutral", "Important", "Very important"}

Page 28

Wat is het maximale bedrag dat u jaarlijks zou willen bijdragen aan de instandhouding van de Zuid-Limburgse natuur?

◯ € 0	◯ € 30	◯ € 100	◯ € 250	◯ Weet ik niet
◯ € 5	◯ € 40	◯ € 120	◯ € 300	
◯ € 10	◯ € 50	◯ € 140	◯ € 350	
◯ € 15	◯ € 60	◯ € 160	◯ € 400	
◯ € 20	◯ € 80	◯ € 200	◯ Meer dan € 400	

Volgende

English translation:

What is the maximum amount you would like to contribute annually to the conservation of nature in Zuid-Limburg?

{The last two options read "More than €400" and "I don't know"}

Page 29

Aan welke van deze betalingsmogelijkheden geeft u de meeste voorkeur wanneer u bijdraagt aan de instandhouding van de Zuid-Limburgse natuur?

◯ Eenmalige bijdrage

◯ Jaarlijkse bijdragen voor een beperkte periode

◯ Jaarlijkse bijdragen voor onbepaalde tijd tot de instandhoudingsdoelstellingen zijn bereikt

◯ Anders, graag toelichten: ____

Volgende

English translation:

Which of these payment options do you prefer when contributing to the conservation of nature in Zuid-Limburg?

-One-time contribution

-Annual contributions for a limited period

-Annual contributions for an indefinite period until conservation objectives are achieved

-Other, please explain: …

Page 30

Aan welke vorm van betaling geeft u de meeste voorkeur?

- ◯ Vrijwillige bijdrage aan een door de overheid beheerd fonds
- ◯ Vrijwillige bijdrage aan een niet-gouvernementele milieuorganisatie (NGO)
- ◯ Extra belasting bestemd voor natuurbehoud
- ◯ Anders, graag toelichten: ______

Volgende

English translation:

What form of payment do you most prefer?

-Voluntary contribution to a government-managed fund

-Voluntary contribution to a non-governmental environmental organization (NGO)

-Additional tax intended for nature conservation

-Other, please explain: …

Page 31

Wie moet volgens u de verantwoordelijkheid nemen voor het behoud van de natuur in Zuid-Limburg? (kruis alles aan wat van toepassing is)

- ☐ Regeringen (nationaal/provinciaal/gemeentelijk)
- ☐ Internationale instanties (bv. Verenigde Naties, Wereldbank)
- ☐ Ngo's en non-profitorganisaties
- ☐ Gemeenschappen (communities)
- ☐ Particulieren
- ☐ Waterschappen
- ☐ Staatsbosbeheer
- ☐ Toeristische sector
- ☐ Particuliere bedrijven
- ☐ Boeren
- ☐ Anders, graag toelichten: ______
- ☐ Geen

Volgende

English translation:

Who do you think should take responsibility for the conservation of nature in Zuid-Limburg? (check all that apply)

-Governments (national/provincial/municipal)

-International bodies (e.g. United Nations, World Bank)

-NGOs and non-profit organizations

-Communities

-Private individuals

-Water boards

-Forestry sector

-Tourism sector

-Private companies

-Farmers

-Other, please explain: …

-None

Page 32 (if respondent selects second option skip to page 34)

Heeft u in de afgelopen 12 maanden ooit geld gedoneerd aan een milieudoel?

- Ja
- Nee

Volgende

English translation:

Have you donated money to an environmental cause in the past 12 months?

-Yes

-No

Page 33

Hoeveel geld heeft u ongeveer besteed aan een milieudoel in de afgelopen 12 maanden (indien u het niet weet, laat het dan open)?

€ [ ]

Volgende

English translation:

Approximately how much money have you spent on an environmental cause in the past 12 months (if you don't know, leave blank)?

€ ...

Page 34 (if respondent selects first or second option skip to page 36)

Wij zijn ook geïnteresseerd in uw gedachten over klimaatverandering.

Welke van de volgende stellingen beschrijft het best uw gedachten over klimaatverandering?

- ◯ Ik denk niet dat het klimaat verandert
- ◯ Ik heb geen idee of het klimaat verandert of niet
- ◯ Ik denk dat het klimaat verandert, maar het is gewoon een natuurlijke schommeling in de temperaturen op aarde
- ◯ Ik denk dat het klimaat verandert, en dat de mens dat grotendeels veroorzaakt

Volgende

English translation:

We are also interested in your thoughts on climate change.

Which of the following statements best describes your thoughts about climate change?

-I don't think the climate is changing

-I have no idea whether the climate is changing or not

-I think the climate is changing, but it's just a natural fluctuation in temperatures on Earth

-I think the climate is changing, and that humans are largely causing it

Page 35

In hoeverre denkt u dat menselijke activiteiten bijdragen aan klimaatverandering, als percentage van de totale klimaatverandering?

- 0%
- 1% tot 20%
- 21% tot 40%
- 41% tot 60%
- 61% tot 80%
- Meer dan 80%
- Ik weet het niet

Volgende

English translation:

To what extent do you think human activities contribute to climate change, as a percentage of total climate change?

-0%

-1% to 20%

-21% to 40%

-41% to 60%

-61% to 80%

-More than 80%

-Don't know

Page 36

Hoe bezorgd bent u in uw dagelijks leven over de volgende onderwerpen?

	Ik denk er nooit over na	Ik denk erover na, maar ik ben helemaal niet bezorgd	Ik ben een beetje bezorgd	Ik ben gematigd bezorgd	Ik ben ernstig bezorgd
Klimaatverandering	○	○	○	○	○
Het uitsterven van dier- en/of plantensoorten	○	○	○	○	○
De 'stikstofcrisis'	○	○	○	○	○
Massatoerisme	○	○	○	○	○
Vervuiling	○	○	○	○	○
Toename in bebouwd gebied	○	○	○	○	○
Intensivering van de landbouw	○	○	○	○	○
Verstedelijking	○	○	○	○	○

English translation:

In your everyday life, how concerned do you feel about the following issues?

{Various issues are listed in the first column: "Climate change", "The extinction of animal and/or plant species", "The 'nitrogen crisis'", "Mass tourism", "Pollution", "Increase in built-up area", "Intensification of agriculture", "Urbanization". Options listed as follows: "I never think about it", "I think about it but I'm not at all concerned", "I'm a little bit concerned", "I'm moderately concerned", "I'm seriously concerned"}

Page 37

Hierna volgen wat algemene vragen.

Volgende

English translation:

Below are some general questions.

Page 38

Denkt u in het algemeen dat geld dat aan de bescherming van natuur in Zuid-Limburg wordt geschonken, goed zal worden besteed of denkt u dat het geld niet goed zal worden besteed?

Gebruik hierbij een schaal van 0 tot 10, waarbij een 0 betekent dat u denkt 'het geld zal niet goed worden besteed', en een 10 dat u denkt 'het geld zal goed worden besteed'. U kunt ook tussenliggende waarden invullen om aan te geven waar op de schaal u zich bevindt.

0 (het geld zal niet goed worden besteed)

10 (het geld zal goed worden besteed)

Volgende

English translation:

In general, do you think that money donated to nature conservation in Zuid-Limburg will be well spent or do you think that the money will not be well spent?

Use a scale of 0 to 10, where 0 means you think 'the money will not be well spent', and 10 means you think 'the money will be well spent'. You can also fill in intermediate values to indicate where you are on the scale.

0

(the money will not be well spent)

10

(the money will be well spent)

Page 39

In het algemeen, in hoeverre bent u bereid om risico's te nemen?

Gebruik hierbij een schaal van 0 tot 10, waarbij een 0 betekent dat u denkt 'totaal niet bereid om risico's te nemen', en een 10 dat u denkt 'totaal bereid om risico's te nemen'. U kunt ook tussenliggende waarden invullen om aan te geven waar op de schaal u zich bevindt.

0 (totaal niet bereid om risico's te nemen)

10 (totaal bereid om risico's te nemen)

Volgende

English translation:

In general, how willing are you to take risks?

Use a scale of 0 to 10, where 0 means you feel 'not at all willing to take risks', and 10 means you feel 'completely willing to take risks'. You can also fill in intermediate values to indicate where you are on the scale.

0

(not at all willing to take risks)

10

(completely willing to take risks)

Page 40

Hoe bereid bent u om iets op te geven wat nu voordelig voor u is om daar in de toekomst meer profijt van te hebben?

Gebruik hierbij een schaal van 0 tot 10, waarbij een 0 betekent dat u denkt 'totaal niet bereid om dit te doen', en een 10 dat u denkt 'totaal bereid om dit te doen'. U kunt ook tussenliggende waarden invullen om aan te geven waar op de schaal u zich bevindt.

0
(totaal niet bereid om dit te doen)

10
(totaal bereid om dit te doen)

Volgende

English translation:

How willing are you to give up something that is beneficial for you today in order to benefit more from that in the future?

Use a scale of 0 to 10, where 0 means you are 'completely unwilling to do so' and 10 means you are 'very willing to do so'. You can also enter values in between to indicate where you are on the scale.

0

(completely unwilling to do so)

10

(very willing to do so)

Page 41

Sommige mensen hebben het gevoel dat zij hun leven volledig onder controle hebben, terwijl andere mensen het gevoel hebben dat wat zij doen geen echte invloed heeft op wat er met hen gebeurt.

Geef op een schaal van 0 tot 10, waarbij 0 betekent 'helemaal geen' en 10 betekent 'zeer veel', aan hoeveel controle u denkt te hebben over de manier waarop uw leven verloopt. U kunt ook tussenliggende waarden invullen om aan te geven waar op de schaal u zich bevindt.

0
(helemaal geen)

10
(zeer veel)

Volgende

English translation:

Some people feel like they have complete control over their lives, while other people feel like what they do has no real impact on what happens to them.

On a scale of 0 to 10, where 0 means 'none at all' and 10 means 'a great deal', indicate how much control you think you have over the way your life turns out. You can also enter values in between to indicate where you fall on the scale.

0

(none at all)

10

(a great deal)

Page 42

Hoe zou u uw politieke ideologie omschrijven (gebruik hiervoor de schuifbalk)?

Zeer liberaal — Zeer conservatief

- [ ] Ik geef er de voorkeur aan mijn politieke ideologie niet te beschrijven

Op welke partij zou u bij de volgende landelijke verkiezingen stemmen?

- ◯ Volkspartij voor Vrijheid en Democratie (VVD)
- ◯ Democraten 66 (D66)
- ◯ Partij voor de Vrijheid (PVV)
- ◯ Christen-democratisch Appèl (CDA)
- ◯ Socialistische Partij (SP)
- ◯ Partij van de Arbeid (PvdA)
- ◯ GroenLinks (GL)
- ◯ Partij voor de Dieren (PvdD)
- ◯ ChristenUnie (CU)
- ◯ Forum voor Democratie (FVD)
- ◯ JA21 (JA21)
- ◯ Staatkundig Gereformeerde Partij (SGP)
- ◯ DENK (DENK)
- ◯ Volt Nederland (Volt)
- ◯ BoerBurgerBeweging (BBB)
- ◯ BIJ1 (BIJ1)
- ◯ 50PLUS (50+)
- ◯ Onafhankelijke Poltiek Nederland (OPNL)
- ◯ Anders
- ◯ Ik geef er de voorkeur aan mijn politieke voorkeur niet kenbaar te maken

Volgende

How would you describe your political ideology (use the slider)?

Very liberal

Very conservative

-I prefer not to describe my political ideology

Which party would you vote for in the next national elections?

-People's Party for Freedom and Democracy (VVD)

-Democrats 66 (D66)

-Party for Freedom (PVV)

-Christian Democratic Appeal (CDA)

-Socialist Party (SP)

-Labour Party (PvdA)

-GreenLeft (GL)

-Party for the Animals (PvdD)

-Christian Union (CU)

-Forum for Democracy (FVD)

-JA21 (JA21)

-Reformed Political Party (SGP)

-THINK (THINK)

-Volt Netherlands (Volt)

-Farmer Citizen Movement (BBB)

-BEE1 (BEE1)

-50PLUS (50+)

-Independent Political Netherlands (OPNL)

-Other: ...

-I prefer not to disclose my political preference

<u>Page 43</u>

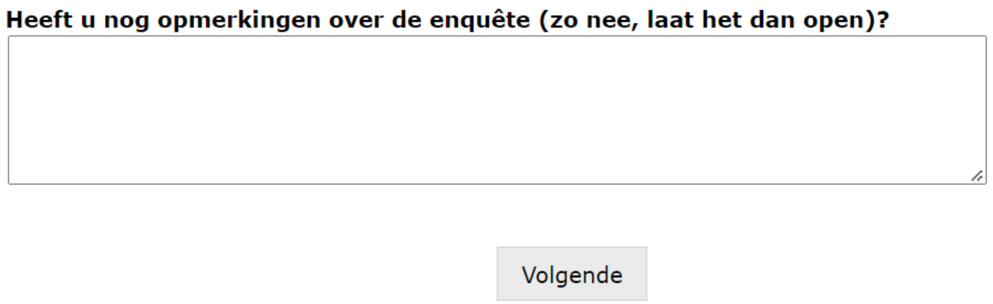

Heeft u nog opmerkingen over de enquête (zo nee, laat het dan open)?

Volgende

English translation:

Do you have any comments about the survey (if not, please leave it open)? ...

References (not in the manuscript)

Barkmann J, Zschiegner AK (2010) Grasslands as a sustainable tourism resource in Germany: environmental knowledge effects on resource conservation preferences. Int J Serv Technol Manag 13:174–191. doi: 10.1504/IJSTM.2010.032076

Barrio M, Loureiro M (2013) The impact of protest responses in choice experiments: an application to a Biosphere Reserve Management Program. For Syst 22:94–105. doi: 10.5424/fs/2013221-03103

Berninger K, Adamowicz W, Kneeshaw D, Messier C (2010) Sustainable forest management preferences of interest groups in three regions with different levels of industrial forestry: an exploratory attribute-based choice experiment. Environ Manage 46:117–133. doi: 10.1007/s00267-010-9507-1

Bernues A, Rodríguez-Ortega T, Ripoll-Bosch R, Alfnes F (2014) Socio-cultural and economic valuation of ecosystem services provided by Mediterranean mountain agroecosystems. Plos One 9. doi: 10.1371/journal.pone.0102479

Birol E, Karousakis K, Koundouri P (2006) Using a choice experiment to account for preference heterogeneity in wetland attributes: the case of Cheimaditida wetland in Greece. Ecol Econ 60:145–156. doi: 10.1016/j.ecolecon.2006.06.002

Brahic E, Rambonilaza T (2015) The impact of information on public preferences for forest biodiversity preservation: a split-sample test with choice experiment method. Rev Econ Polit 125:253–275.

Broadbent CD, Grandy JB, Berrens RP (2010) Testing for hypothetical bias in a choice experiment using a local public good: riparian forest restoration. Int J Ecol Econ Stat 19:1–19.

Cai Y, Zhao M, Shi Y, Khan I (2020) Assessing restoration benefit of grassland ecosystem incorporating preference heterogeneity empirical data from Inner Mongolia Autonomous Region. Ecol Indic 117. doi: 10.1016/j.ecolind.2020.106705

Carlsson F, Frykblom P, Liljenstolpe C (2003) Valuing wetland attributes: an application of choice experiments. Ecol Econ 47:95–103. doi: 10.1016/j.ecolecon.2002.09.003

Cerda C, Barkmann J, Marggraf R (2014) Non-market economic valuation of the benefits provided by temperate ecosystems at the extreme south of the Americas. Reg Environ Change 14:1517–1531. doi: 10.1007/s10113-014-0591-2

Colombo S, Hanley N (2008) How can we reduce the errors from benefits transfer? An investigation using the choice experiment method. Land Econ 84:128–147. doi: 10.3368/le.84.1.128

Decker KA, Watson P (2017) Estimating willingness to pay for a threatened species within a threatened ecosystem. J Environ Plann Man 60:1347–1365. doi: 10.1080/09640568.2016.1221797

de Ayala A, Hoyos D, Mariel P (2015) Suitability of discrete choice experiments for landscape management under the European Landscape Convention. J Forest Econ 21:79–96. doi: 10.1016/j.jfe.2015.01.002

de Valck J, Vlaeminck P, Broekx S, Liekens I, Aertsens J, et al (2014) Benefits of clearing forest plantations to restore nature? Evidence from a discrete choice experiment in Flanders, Belgium. Landscape Urban Plan 125:65–75. doi: 10.1016/j.landurbplan.2014.02.006

Dias V, Belcher K (2015) Value and provision of ecosystem services from prairie wetlands: a choice experiment approach. Ecosyst Serv 15:35–44. doi: 10.1016/j.ecoser.2015.07.004

Dissanayake ST, Ando AW (2014) Valuing grassland restoration: proximity to substitutes and trade-offs among conservation attributes. Land Econ 90:237–259. doi: 10.3368/le.90.2.237

Elsasser P, Englert H, Hamilton J (2010) Landscape benefits of a forest conversion programme in North East Germany: results of a choice experiment. Ann For Res 53:37–50. doi: 10.15287/afr.2010.113

Farreras V, Mavsar R (2012) Burned forest area or dead trees? A discrete choice experiment for Catalan citizens. Economía Agraria y Recursos Naturales 12:137–153. doi: 10.7201/earn.2012.02.06

Garrod G, Ruto E, Snowdon P (2009) Assessing the value of forest landscapes: a choice experiment approach. Arboricultural Journal 32:189–211. doi: 10.1080/03071375.2009.9747573

Giergiczny M, Czajkowski M, Żylicz T, Angelstam P (2015) Choice experiment assessment of public preferences for forest structural attributes. Ecol Econ 119:8–23. doi: 10.1016/j.ecolecon.2015.07.032

Glenk K, Martin-Ortega J (2018) The economics of peatland restoration. Journal of Environmental Economics and Policy 7:345–362. doi: 10.1080/21606544.2018.1434562

Hanley N, Colombo S, Mason P, Johns H (2007) The reform of support mechanisms for upland farming: paying for public goods in the severely disadvantaged areas of England. J Agr Econ 58:433–453. doi: 10.1111/j.1477-9552.2007.00114.x

Hasund KP, Kataria M, Lagerkvist CJ (2011) Valuing public goods of the agricultural landscape: a choice experiment using reference points to capture observable heterogeneity. J Environ Plann Man 54:31–53. doi: 10.1080/09640568.2010.502753

Hoehn JP, Lupi F, Kaplowitz MD (2010) Stated choice experiments with complex ecosystem changes: the effect of information formats on estimated variances and choice parameters. J Agr Resour Econ 35:568–590. doi: 10.22004/ag.econ.99121

Horne P, Boxall PC, Adamowicz WL (2005) Multiple-use management of forest recreation sites: a spatially explicit choice experiment. Forest Ecol Manag 207:189–199. doi: 10.1016/j.foreco.2004.10.026

Hoyos D, Mariel P, Pascual U, Etxano I (2012) Valuing a Natura 2000 network site to inform land use options using a discrete choice experiment: an illustration from the Basque Country. J Forest Econ 18:329–344. doi: 10.1016/j.jfe.2012.05.002

Huber R, Hunziker M, Lehmann B (2011) Valuation of agricultural land-use scenarios with choice experiments: a political market share approach. J Environ Plann Man 54:93–113. doi: 10.1080/09640568.2010.502761

Kefale T, Hagos F, van Rooijen D, Haileslassie A (2021) Farmers' willingness to pay for alternative resource management practices in the Bale Eco-Region, Ethiopia: an application of choice experiment. Heliyon 7. doi: 10.1016/j.heliyon.2021.e08159

Mao B, Ao C, Wang J, Xu L (2020) The importance of loss aversion in public preferences for wetland management policies: evidence from a choice experiment with reference-dependent discrete choice model. Wetlands 40:599–608. doi: 10.1007/s13157-019-01195-2

Meyerhoff J, Liebe U, Hartje V (2009) Benefits of biodiversity enhancement of nature-oriented silviculture: evidence from two choice experiments in Germany. J Forest Econ 15:37–58. doi: 10.1016/j.jfe.2008.03.003

Mombo F, Lusambo L, Speelman S, Buysse J, Munishi P, et al (2014) Scope for introducing payments for ecosystem services as a strategy to reduce deforestation in the Kilombero wetlands catchment area. Forest Policy Econ 38:81–89. doi: 10.1016/j.forpol.2013.04.004

Müller A, Olschewski R, Unterberger C, Knoke T (2020) The valuation of forest ecosystem services as a tool for management planning–A choice experiment. J Environ Manage 271. doi: 10.1016/j.jenvman.2020.111008

Naidoo R, Adamowicz WL (2005) Biodiversity and nature-based tourism at forest reserves in Uganda. Environ Dev Econ 10:159–178. doi: 10.1017/S1355770X0400186X

Newell LW, Swallow SK (2013) Real-payment choice experiments: valuing forested wetlands and spatial attributes within a landscape context. Ecol Econ 92:37–47. doi: 10.1016/j.ecolecon.2012.08.008

Nie X, Jin X, Wu J, Li W, Wang H, et al (2023) Evaluation of coastal wetland ecosystem services based on modified choice experimental model: a case study of mangrove wetland in Beibu Gulf, Guangxi. Habitat Int 131. doi: 10.1016/j.habitatint.2022.102735

Nordén A, Coria J, Jönsson AM, Lagergren F, Lehsten V (2017) Divergence in stakeholders' preferences: evidence from a choice experiment on forest landscapes preferences in Sweden. Ecol Econ 132:179–195. doi: 10.1016/j.ecolecon.2016.09.032

Obeng EA, Dakurah I, Oduro KA, Obiri BD (2021) Local communities' preferences and economic values for ecosystem services from Mole National Park in Ghana: a choice experiment approach. Global Ecology and Conservation 32. doi: 10.1016/j.gecco.2021.e01904

Olsen SB (2009) Choosing between internet and mail survey modes for choice experiment surveys considering non-market goods. Environ Resour Econ 44:591–610. doi: 10.1007/s10640-009-9303-7

Pelletier MC, Tocock M, MacDonald DH, Rose JM, Sullivan CA (2022) Does information matter in the value of a wetland? J Environ Plann Man 65:1323–1348. doi: 10.1080/09640568.2021.1995339

Rewitzer S, Huber R, Grêt-Regamey A, Barkmann J (2017) Economic valuation of cultural ecosystem service changes to a landscape in the Swiss Alps. Ecosyst Serv 26:197–208. doi: 10.1016/j.ecoser.2017.06.014

Schaafsma M, Brouwer R, Liekens I, De Nocker L (2014) Temporal stability of preferences and willingness to pay for natural areas in choice experiments: a test–retest. Resour Energy Econ 38:243–260. doi: 10.1016/j.reseneeco.2014.09.001

Senzaki M, Yamaura Y, Shoji Y, Kubo T, Nakamura F (2017) Citizens promote the conservation of flagship species more than ecosystem services in wetland restoration. Biol Conserv 214:1–5. doi: 10.1016/j.biocon.2017.07.025

Shi Y, Li C, Zhao M (2021) Herders' aversion to wildlife population increases in grassland ecosystem conservation: evidence from a choice experiment study. Global Ecology and Conservation 30. doi: 10.1016/j.gecco.2021.e01777

Sinclair M, Sagar MV, Knudsen C, Sabu J, Ghermandi A (2021) Economic appraisal of ecosystem services and restoration scenarios in a tropical coastal Ramsar wetland in India. Ecosyst Serv 47. doi: 10.1016/j.ecoser.2020.101236

Tan Y, Lv D, Cheng J, Wang D, Mo W, et al (2018) Valuation of environmental improvements in coastal wetland restoration: a choice experiment approach. Global Ecology and Conservation 15. doi: 10.1016/j.gecco.2018.e00440

Tu G, Abildtrup J (2016) The effect of experience on choosing where to go: an application to a choice experiment on forest recreation. J Environ Plann Man 59:2064–2078. doi: 10.1080/09640568.2015.1119105

Upton V, Dhubháin ÁN, Bullock C (2012) Preferences and values for afforestation: the effects of location and respondent understanding on forest attributes in a labelled choice experiment. Forest Policy Econ 23:17–27. doi: 10.1016/j.forpol.2012.06.006

Valasiuk S, Giergiczny M, Żylicz T, Klimkowska A, Angelstam P (2018) Conservation of disappearing cultural landscape's biodiversity: are people in Belarus willing to pay for wet grassland restoration? Wetl Ecol Manag 26:943–960. doi: 10.1007/s11273-018-9622-y

Vecchiato D, Tempesta T (2013) Valuing the benefits of an afforestation project in a peri-urban area with choice experiments. Forest Policy Econ 26:111–120. doi: 10.1016/j.forpol.2012.10.001

Weller P, Elsasser P (2018) Preferences for forest structural attributes in Germany–evidence from a choice experiment. Forest Policy Econ 93:1–9. doi: 10.1016/j.forpol.2018.04.013

Westerberg VH, Lifran R, Olsen SB (2010) To restore or not? A valuation of social and ecological functions of the Marais des Baux wetland in Southern France. Ecol Econ 69:2383–2393. doi: 10.1016/j.ecolecon.2010.07.005

Xu S, He X (2022) Estimating the recreational value of a coastal wetland park: application of the choice experiment method and travel cost interval analysis. J Environ Manage 304. doi: 10.1016/j.jenvman.2021.114225
